# Insights into the Chemical Exposome during Pregnancy: A Non-Targeted Analysis of Preterm and Term Births

**DOI:** 10.1021/acs.est.4c08534

**Published:** 2024-11-11

**Authors:** Xiaowen Ji, Mathusa Lakuleswaran, Whitney Cowell, Linda G. Kahn, Marina Sirota, Dimitri Abrahamsson

**Affiliations:** † Division of Environmental Pediatrics, Department of Pediatrics, Grossman School of Medicine, New York University, New York, New York 10016, United States; ‡ Bakar Computational Health Sciences Institute, UCSF, San Francisco, California 94158, United States; § Department of Obstetrics, Gynecology and Reproductive Sciences, University of California, San Francisco, San Francisco, California 94158, United States; ⊥ Department of Pediatrics, University of California, San Francisco, San Francisco, California 94158, United States

**Keywords:** non-targeted analysis, high-resolution mass spectrometry, preterm birth, exogenous chemicals, exposure, chemical associations

## Abstract

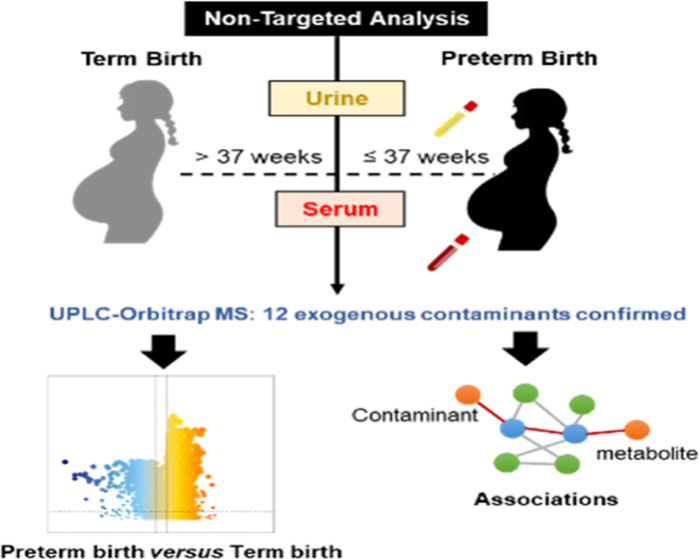

Human-made chemicals are ubiquitous, leading to chronic exposure to complex mixtures of potentially harmful substances. We investigated chemical exposures in pregnant women in New York City by applying a non-targeted analysis (NTA) workflow to 95 paired prenatal urine and serum samples (35 pairs of preterm birth) collected as part of the New York University Children’s Health and Environment Study. We analyzed all samples using liquid chromatography coupled with Orbitrap high-resolution mass spectrometry in both positive and negative electrospray ionization modes, employing full scan and data-dependent MS/MS fragmentation scans. We detected a total of 1524 chemical features for annotation, with 12 chemicals confirmed by authentic standards. Two confirmed chemicals dodecyltrimethylammonium and *N*,*N*-dimethyldecylamine *N*-oxide appear to not have been previously reported in human blood samples. We observed a statistically significant differential enrichment between urine and serum samples, as well as between preterm and term birth (*p* < 0.0001) in serum samples. When comparing between preterm and term births, an exogenous contaminant, 1,4-cyclohexanedicarboxylic acid (tentative), showed a statistical significance difference (*p* = 0.003) with more abundance in preterm birth in serum. An example of chemical associations (12 associations in total) observed was between surfactants (tertiary amines) and endogenous metabolites (fatty acid amides).

## Abstract 2

Non-targeted analysis of urine and serum samples from pregnant women reveals a potential link between environmental contaminants and preterm birth.

## 1 Introduction

Human beings are already exposed to various synthetic chemicals through exposure to consumer products, packaged and processed food, contaminated drinking water, and polluted air, and the number is only increasing.^1^ Many of these chemicals may be adsorbed by the human body and potentially pose a threat to human health. In addition new compounds, also known as transformation products, might form through biotic and abiotic processes when these chemicals are exposed to different environments.^2,3^ Approximately 350,000 registered chemical substances have been used for commercial production and use over the past 40 years across 19 countries and regions.^4^ Moreover, the United States Environmental Protection Agency (US EPA) has listed over 1,218,248 chemicals of environmental importance on EPA’s CompTox Chemicals Dashboard (https://comptox.epa.gov/dashboard/). Recent estimates suggest that only 10% of chronic human diseases can be attributed to genetics, leaving 90% potentially related at least in part to environmental factors.^5^

Pregnant women are routinely exposed to human-made chemicals from the ambient environment that may result in adverse outcomes for both the mother and fetus. Previous studies have highlighted that maternal exposure to environmental contaminants can increase the risk of obesity,^6^ asthma,^7^ and various conditions in offspring, including preterm birth.^8^ The timing of exposure is also an important factor as the effects of an exposure likely depend on the developmental processes that it coincides with. Epidemiological evidence indicates that exposure to environmental contaminants at any time between preconception and birth can restrict fetal growth, resulting in a fetus not reaching its full growth potential (lower birth weight than expected).^9^ The fetal brain is particularly susceptible to prenatal exposure to endocrine-disrupting chemicals, as neurulation and neuronal proliferation begin within the first trimester, while other processes such as neural migration, myelination, synaptogenesis, and apoptosis start midgestation and continue rapidly until birth.^10,11^ Investigating chemical exposure during the critical windows can provide insight on underlying biological mechanisms.

Traditional monitoring of contaminants in human samples relies on prior hypotheses, the availability of analytical standards, and the existence of a validated chromatographic method. Approximately 450 environmental chemicals are regularly measured in human samples (e.g., whole blood, serum, and urine) by the US National Health and Nutrition Examination Survey (NHANES).^12^ This only accounts for approximately 0.5 and 0.04% of chemicals listed under a US federal law of Toxic Substances Control Act (TSCA) and EPA’s CompTox Chemicals Dashboard, respectively. Such conventional approaches cannot capture the totality of chemical exposures and consequently important associations with various health outcomes may be missed. The advancements in high-resolution mass spectrometry (HRMS) have improved our ability to analyze thousands of different chemicals in a single run due to its high resolving power (>30,000 fwhm at *m*/*z* 200), mass accuracy (1–5 ppm), and high scan speed (12 Hz).^13^ Combined with a preseparation technique such as gas or liquid chromatography (GC/LC), HRMS shows great promise in detecting unknown chemicals across various domains.^14^ In recent years, non-targeted analysis (NTA) using HRMS has successfully been used to screen human samples, resulting in the discovery of numerous exogenous compounds (e.g., pesticide metabolites, endocrine-disrupting compounds, and poly- and perfluoroalkyl substances).^15−17^ Numerous studies for unknown compounds have focused on the possible compounds that were postulated with suspect lists (suspect screening analysis, SSA).^18^ However, in NTA, no such suspect lists exist for unknown compounds. There is currently a great need for the application of NTA to characterize different pathways of exposures in public health studies.

Based on previous NTA methods,^17,19,20^ we developed a workflow to comprehensively profile all detectable chemical exposures and metabolites in biospecimens from a racially and socioeconomically diverse sample of pregnant women from New York City. The aims of this study were 3-fold: (1) to analyze 95 paired serum and urine samples from pregnant women using NTA and study their chemical exposures, (2) characterize differences in chemical enrichment between urine and serum, within each biospecimen type, between preterm and term births, and (3) explore the associations of endogenous metabolites with exogenous chemicals.

## 2 Materials and Methods

### 2.1 Non-targeted Analysis Workflow

The NTA workflow contained three major steps: (1) sample treatment and chemical analysis, (2) data cleansing and processing, and (3) data analysis (Figure S1, Supporting Information). In this work, the individual samples and pooled samples were aimed to obtain MS^1^ and MS^1^/MS^2^ spectra, respectively. We used MS^1^ data from the individual samples to examine the statistical differences in chemical enrichment between different groups of samples, MS^1^/MS^2^ spectra (fragments ≥2) from pooled samples to match available databases composed of authentic standards and in silico predicted spectra, and to match to authentic standards in our laboratory. The chemical abundances in the diluted urine samples were adjusted using the creatinine normalization approach (Details in Text S1, Supporting Information). Chemical identifications and annotations were ranked based on the system proposed by Schymanski et al.^21^ (details in Text S2). After annotating the chemical features from MS/MS data from the pooled samples, the annotation information was merged with the MS^1^ alignment from the individual samples, based on retention time (within 0.1 min) and MS^1^ mass accuracy (∼2 ppm).

Considering the complexity and heterogeneous components in the present samples, different methods and tools were applied to explore and analyze the MS data. Following this workflow, we first used MS-DIAL to export the MS data for statistical analysis and MS/MS database matching. Python was used as the programing language for data analysis. All python scripts are available on GitHub at the following link: https://github.com/jixiaowen4321/Jixiaowen. We also applied Thermo FreeStyle 1.8 for ion peak identification and Compound Discoverer 3.2 for matching with the Thermo mzCloud database. The basic parameters of MS-DIAL and Compound Discoverer are shown in Text S3. The details of each step in this workflow are described in the sections below.

### 2.2 Study Participants Information

For this study we used paired urine and serum samples collected between 2020 and 2022 during the same prenatal study visit from 95 participants in the New York University Children’s Health and Environment Study (NYU CHES). NYU CHES is an ongoing pregnancy and birth cohort study that has been recruiting pregnant patients ≥18 years of age and <18 weeks of gestation from NYU Langone Health-affiliated hospitals since March, 2016. The samples were mostly collected in the first trimester with 2 and 5 pairs for the second and third trimesters, respectively. The ethnic groups of participants include Hispanic, non-Hispanic White, non-Hispanic Black, Asian, mixed race, and other. Participant characteristics are presented in Table 1. All samples were stored in bisphenol A- and phthalate-free polypropylene tubes at −80 °C.

**Table 1:** Characteristics of NYU CHES Participants Included in This Analysis (*N* = 95)

demographic parameters	value
Participant’s Race/Ethnicity *n* (%)	
hispanic	31.1
non-hispanic white	43.4
non-hispanic black	2.8
asian	18.9
other	1.9
mixed race	1.9
Prepregnancy Body Mass Index (BMI, kg/m^2^)	
underweight (BMI < 18.5), %	3.8
normal weight (BMI = 18.5–25), %	58.1
overweight (BMI = 25–30), %	25.7
obesity (BMI > 30), %	12.4
Maternal Education^a^ (%)	
high school or less	26.0
some college but no degree	6.0
associate degree	4.0
bachelor’s degree	28.0
postgraduate degree	36.0
missing	6.0
Income^a^ (%)	
<$30,000	12.2
$30,000–$49,999	8.2
$50,000–$74,999	10.2
$75,000–$99,999	2.0
≥$100,000	49.0
missing value	24.5
number of preterm births	35
maternal age at enrollment (years), mean (std)	31.6 (5.1)
prepregnancy weight (kg), mean (std)	64.5 (16.3)
maternal height (cm)	161.2 (7.4)
gestational age (weeks), mean (std)	38.3 (2.3)
smoking^a^ (%)	1.9
alcohol use during pregnancy^a^ (%)	11.9
missing value (%)	2.8

a When a parameter has missing data, it means that the participant chose the option “Prefer not to answer”/“Don’t Know” from the questionnaires. The values in the parentheses correspond to the unit in the column of demographic parameters. Std indicates the standard deviations.

### 2.3 Sample Preparation and Analysis

All samples were completely thawed at room temperature (∼21 °C) and homogenized using a vortex mixer before extraction. For individual samples, 100 μL of sample was pipetted into a microcentrifuge tube. For pooled samples, 15 pools each of serum and urine were constructed from 10 individual 20 μL samples (200 μL total) randomly selected based on sample IDs using Python’s random.choices() method. For extraction, 400 μL methanol was added to the tube, which was then shaken using a vortex mixer and centrifuged at 5000 rpm for 10 min. The upper clear layer of methanol was immediately filtered into an autosampler vial with an insert using a nylon membrane (pore size: 0.2 μm, Phenomenex, Torrance, CA). Triplicates of HPLC water were used as laboratory blanks and followed the same sample preparation procedure. A Vanquish UHPLC coupled with an Orbitrap Exploris 240 mass spectrometer (Thermo, MA) was used for the chemical analysis. The details of the instrument method and QA/QC are provided in Texts S4 and S5 and Spreadsheet S1, respectively.

### 2.4 Chemical Annotations and Source Attributions

To confirm the chemicals in our samples, all data were first matched by the databases containing MS^1^ and MS^2^ from authentic standards, i.e., MS-DIAL metabolomics, MassBank of North America, Massbank Europe, and mzCloud. These databases (mzCloud via Compound Discoverer) were downloaded in “.msp” format for import into MS-DIAL. Afterward, the sources of compounds were attributed by searching the ChemSpider database (http://www.chemspider.com/), Blood Exposome Database (BED, https://bloodexposome.org/), Human Metabolome Database (HMD, https://hmdb.ca/), EPA CompTox Chemicals Dashboard (https://comptox.epa.gov/dashboard/).

### 2.5 Data Cleansing and Processing

All data processing was done using Python (version 3.11.5) as the programming language and the following packages for data handling, data analysis and visualizations: pandas, numpy, matplotlib, seaborn, and scipy. The scripts were written using the JupyterLab and Spyder interfaces.

#### 2.5.1 Imputation and Batch Effects

Before data analysis, the data set was processed for imputation of missing data and batch correction. We first calculated the frequency of each chemical feature among the samples and selected a detection frequency of 70% as the cutoff for imputation (Sensitivity analysis of 60, 70, and 80% cutoff for imputation did not alter the results). The background noise for the Orbitrap was set as the minimum peak area (≤10,000). To fill in the data points below the MDLs, we used a previously developed imputation method.^17^ This method fills the missing data points using the standard deviation and median calculated from the measured data for each chemical feature, and adjusts the filled data to fit a normal distribution.

Due to variations in sample preparation and liquid chromatography-mass spectrometry (LC-MS) conditions across the four batches in our instrumental run, technical effects may obscure the true biological differences in our samples. To minimize systematic differences between batches, urine samples were analyzed alongside their corresponding serum samples, with all samples randomly positioned within the sequence. The remaining batch effects were corrected using a batch correction package called “ComBat”. This package employs parametric and nonparametric Bayes methods for adjusting data for batch effects. The details of the batch correction method have been described in the study of Johnson et al.^22^ The “ComBat” package can partially remove these batch effects for HRMS data, allowing us to analyze the biological differences between various samples.

#### 2.5.2 Data Analysis

##### 2.5.2.1 Unsupervised Clustering

We conducted a principal component analysis (PCA) to examine the differences before and after “Combat” batch correction among four batches. We also conducted a correlation analysis for the correlation of the PCs 1–3 with sample type and batch.

The differences for groups of similar data points of chemical composition between urine and serum samples, and between preterm birth and term birth samples were evaluated by employing hierarchically clustered heatmap using the Seaborn Python package.^23^

##### 2.5.2.2 Relationships of Chemical Features in Different Sample Types

The relative abundance and detected percentage were used to explore the relationships of chemical features between urine and serum samples. The abundance was first log-transformed and then averaged across all 95 samples for each chemical feature. The average values were used for the linear regression model to examine the correlation between urine and serum samples.

We used a volcano plot of average areas of chemical features to assess statistical significance through a *t* test and the magnitude of change between preterm birth and term birth samples (in serum and urine, respectively), as well as between serum and urine samples. This approach helps identify chemicals that differ significantly between preterm and term birth samples and between serum and urine samples.

##### 2.5.2.3 Molecular Network Analysis for Different Annotated Chemicals

After annotating chemical features as described in Section 2.4, Pearson correlations between chemicals annotated as endogenous metabolites and all other annotated chemicals were used for molecular network analysis. In this study, the network indicates the association between chemical features. The purpose of the network is to visualize the inter and intramolecular associations between endogenous metabolites and other chemical features, including exogenous contaminants. For the visualization, d3.js was used to show the networks for relationships between endogenous metabolites and other chemicals. Because of the large number of associations in the complex network, we only focused on the Levels 1 and 2 compounds with an absolute correlation coefficient (*R*) > 0.5 and revisualized the networks based on these chemicals.

##### 2.5.2.4 Statistical Analyses

For conducting correlations, we used Pearson’s *R*, and for statistical differences between two groups (e.g., preterm and term birth), we used a *t*-test. The *p*-values were adjusted using the Benjamini–Hochberg test with a null hypothesis of 5% false positives.

## 3 Results

### 3.1 Filtering Chemical Features

After the alignment of 4 batches, the total amount of chemical features in both urine and serum samples (*n* total = 190 samples) without cleanup processing from the full scan was 112,737 for ESI^+^ and 82,335 for ESI^–^ (Figure S2a,b). After eliminating the features that were adducts that were linked to other ion(s) and detection frequency below 70%, the processed data set was decreased to 21,952 features for ESI^+^ and 10,006 features for ESI^–^. By merging the ESI^+^ and ESI^–^ data sets (±monoisotopic H: 1.00782), the pair of 2219 features in both ESI^+^ and ESI^–^ was observed based on the RT time difference <0.5 min and mass difference ≤5 ppm.

The chemical features in the data set from the pooled samples run by full scan/ddMS2 (ESI^+^: 46,228; ESI^–^: 27,252; Figure S2c,d) were reduced by filtering the ions with product ions, resulting in 1,524 features (Levels ≥3).

### 3.2 Batch Correction

From our data set, we observed systematic variations arising from differences between batches or groups. For example, the data set without batch correction for serum samples, no clusters of PC1 and PC2 loadings were observed for preterm and term birth sample types (Figure S3a-1). After batch correction, two distinct clusters corresponding to preterm and term birth samples were observed in serum (Figure S3a-2). No batch effect was observed after correction (Figure S3a-4), compared to the four distinct clusters of PC1 and PC2 loadings before correction (Figure S3a-3). After batch correction, the significant differences were found between PC1 and preterm–term birth sample types (*p* < 0.01), as well as between PC2 loadings and preterm–term birth samples (*p* < 0.01) (Figure S4a,b).

In urine samples, clusters of PC1 and PC2 loadings for preterm and term birth were not separated before batch correction (Figure S3b-1), and were only partially separated after batch correction (Figure S3b-2). The batch effect in the four batches of urine samples was not pronounced before correction (Figure S3b-3) and was absent after correction (Figure S3b-4). After batch correction, a significant difference could be observed between PC1 loadings and preterm–term birth sample types (*p* < 0.01) ( Figure S4c,d).

In the combined serum and urine data set after batch correction, PC1 and PC2 loadings were able to separate serum and urine samples, though some data points were not well separated (Figures S3c-1 and 2). For the four batches, the batch effect was not strong in the combined data set before correction (Figure S3c-3) and was eliminated after correction (Figure S3c-4). After batch correction, the significant differences were observed between PC1 loadings and sample type or batches (*p* < 0.01), as well as between PC3 loadings and sample type or batches (*p* < 0.01) (Figure S4e,f).

### 3.3 Chemical Annotation

The processed chemical features merged from ESI^+^ and ESI^–^ modes were used for database matching. Out of 1524 chemical features with MS^2^ information, we were able to annotate 344 features, with a match score of over 90% using MS-DIAL and Compound Discoverer, and 18 features were found to be common in both ESI^+^ and ESI^–^ (Spreadsheet S2).

It is critical to discern whether the detected compounds are exogenous or endogenous, especially those expected in urine and serum samples. Many compounds enter the human body through food ingestion (e.g., nutrients and natural products) and drugs (including intermediate chemicals during pharmaceutical production) and their derivatives. The metabolic processes in the human body create a plethora of transformation products from the parent compounds. A challenge that we encountered when trying to attribute sources to the detected compounds was that compounds often have multiple uses and can be both endogenous and exogenous.^24^ Another challenge when dealing with chemical databases related to the human exposome is that, in many cases, only the monoisotopic mass of the chemical is available for matching, and the MS^2^ spectra are missing.

We compiled information from multiple sources to reflect whether the compounds are intentionally ingested and whether they are industrial or natural products. The integrated data of identified compounds (Levels 1 and 2) are listed in Supporting Information Spreadsheet S2, where we present five categories of sources and uses:

(1) Endogenous Metabolites: Substances naturally produced from human issues during the metabolism process.
(2) Natural Products: substances derived from food or nutrients.
(3) Drugs: Substances intentionally ingested by people for different treatments, such as therapeutics/prescription drugs.
(4) Personal Care Products (PCPs): Substances used in cosmetics or other personal care products.
(5) Exogenous Contaminants: Substances present in human working/living environments, such as additives in house furnishings.

If there was no source indicated, the source of the compound was marked as “unknown”. While it is generally expected that one compound will be attributed to one category, it is often the case that one compound can have multiple sources. For example, d-camphor (CAS: 464–48–2) was attributed to several sources because it is a constituent of various foods, medicines (such as treatment of colds and topical analgesics), and various cosmetics in the US. Some derivatives were annotated based on their parent compounds. The classification of the 327 chemicals was as follows: endogenous metabolites (203), exogenous contaminants (96), drugs (101), natural products (38), and PCPs (45) (Figure S5).

From the analytical standards in our laboratory (70 standards, Spreadsheet S3), 12 chemicals were confirmed by comparing RT (<0.05 min), and precursor ion/product ions (<5 ppm) (example shown in Figure S6). These included two organophosphorus compounds (triisobutyl phosphate and tributyl phosphate), five amines (triisopropanolamine, tributylamine, diphenylamine, dodecyltrimethylammonium, and *N*,*N*-dimethyldecylamine *N*-oxide), three phenol derivatives (4-nitrophenol, 3-aminophenol, and 2-aminophenol), propiconazole, and 2,2,6,6-tetramethyl-4-piperidinol (Spreadsheet S3). Most chemicals with MS^2^ data were categorized as Level 4 (unknown, with no database match observed) (Figure S7).

**Figure 1:**
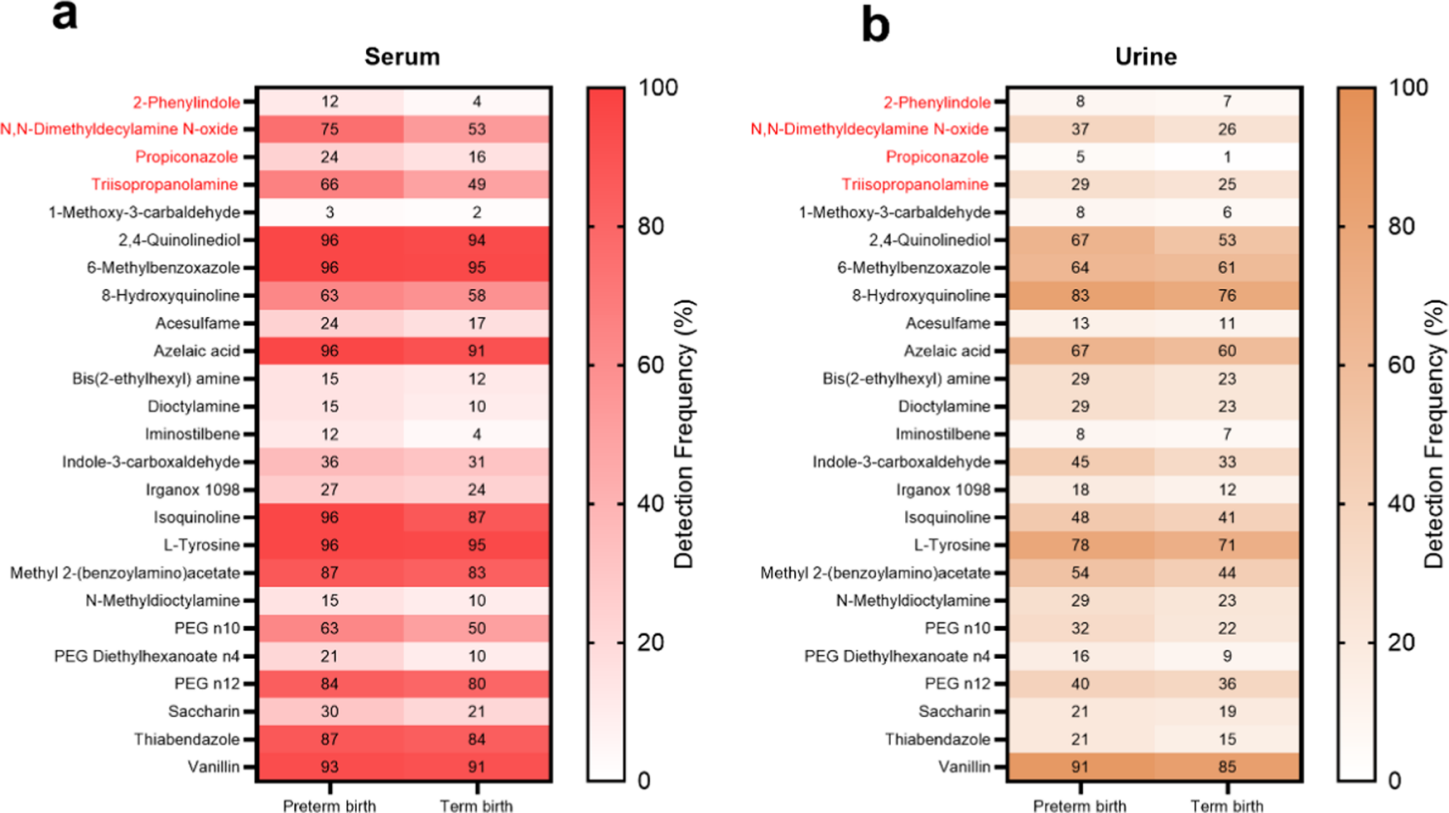
Detection frequency (%) of annotated chemicals (Levels 1 and 2) classified as exogenous contaminants in preterm and term birth samples: chemicals with higher detection frequency in preterm birth for both serum: (a) and urine (b). The chemical names in red represent the confirmed chemicals (Level 1) by the authentic standards.

We observed that all exogenous contaminants (Levels 1–2) were detected more frequently in preterm birth samples in both serum and urine (Figures 1a,b and S8a,b). The detection frequency of all annotated exogenous contaminants is shown in Figure S9. We found that four confirmed chemicals (2-phenylindole, *N*,*N*-dimethyldecylamine, propiconazole, and triisopropanolamine) were detected more frequently in preterm birth samples, in both serum and urine (Figure 1).

### 3.4 Data Analysis

#### 3.4.1 Difference between Preterm and Term Birth

**Figure 2:**
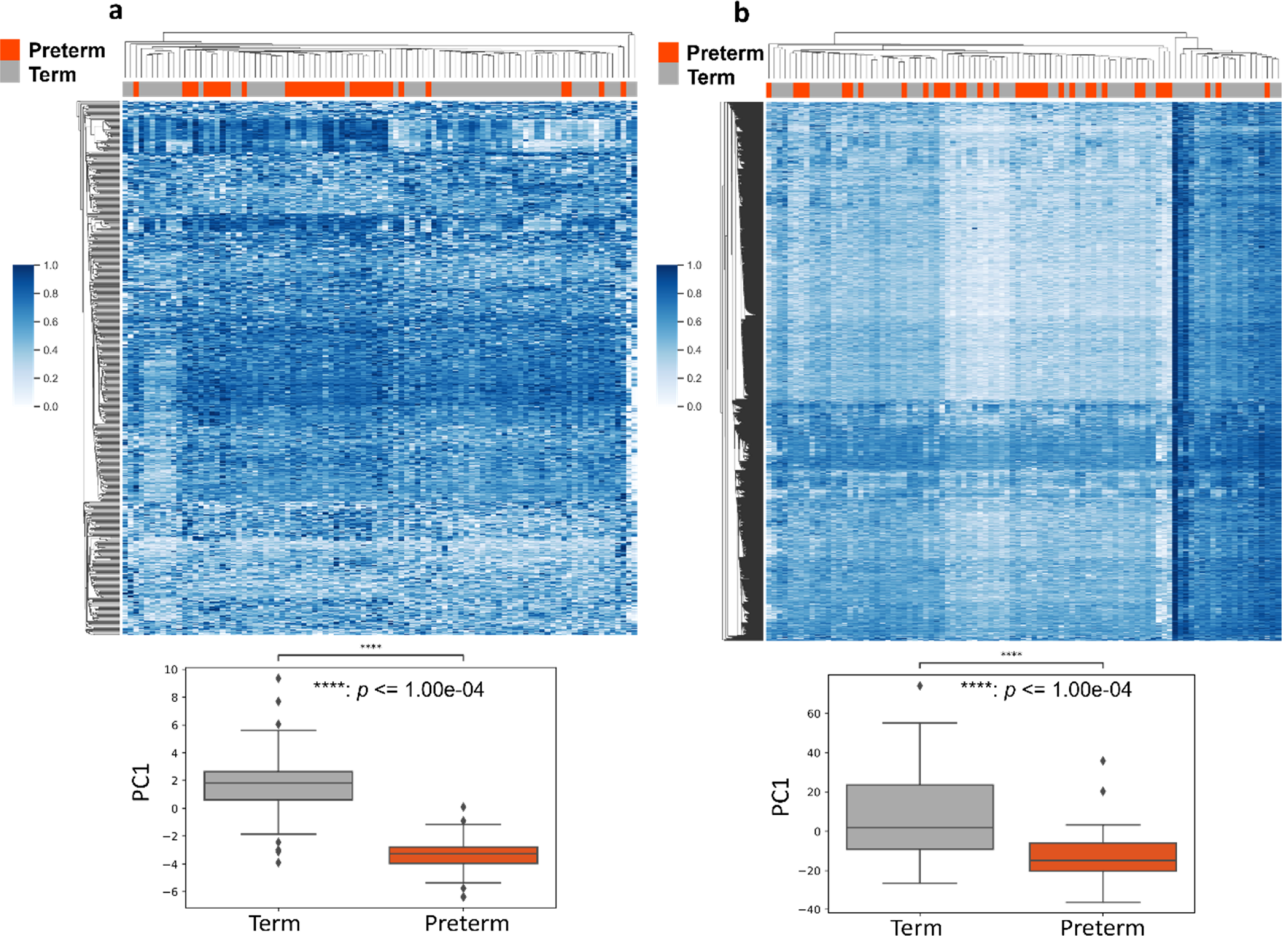
Clustering heatmap after batch effect correction for serum and urine samples. The chemical features reveal the differential enrichment in preterm versus term births among serum samples (a) and urine samples (b) after multiple testing correction (Benjamini-Hochberg test, 5% false discovery rate). The bottom and top of the boxes represent the 25th and 75th percentiles, the error bars denote the 10th to 90th percentiles, and the solid line indicates the median value.

**Figure 3:**
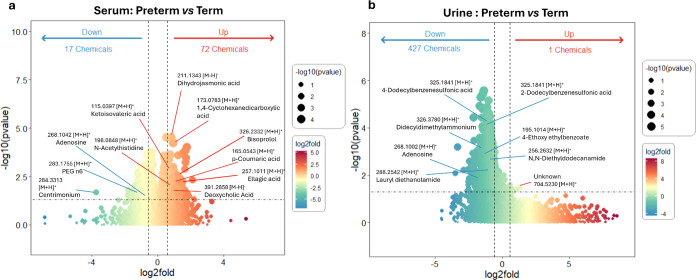
Volcano plot of the log-transformed ratios and corresponding p-values of chemical features with a cutoff frequency of 70% from ESI^+^ and ESI^–^ modes illustrates the data: the statistical differences in chemical features between preterm births and term births in serum (a) and urine (b). The horizontal dashed line indicates the cutoff for the log *p*-value (*p* < 0.05), and the vertical dashed lines indicate the cutoff for fold change (log_2_ fold change = 1.2).

In serum, clusters of different chemicals’ enrichment were observed between preterm and term birth samples (Figure 2a). The statistical differences in PC1 loadings between preterm and term birth samples were significant after batch correction (*p* < 0.0001). Among the 1547 significantly different LC-MS features between preterm and term birth samples (*p* < 0.05), 3 out of 17 chemicals from the downregulated area (log_2_ fold < −1.2) and 8 out of 72 chemicals from the upregulated area (log_2_ fold >1.2) could be tentatively annotated (Spreadsheet S4). For example, the annotated chemicals in the downregulated area have poly(ethylene glycol) (PEG) n6 (*m*/*z*: 283.1755 [M + H^+^]) and centrimonium (*m*/*z*: 284.3313 [M + H^+^]) (Figure 3a). Those in the upregulated area have *N*-acetylhistidine (*m*/*z*: 198.0848 [M + H^+^]), and deoxycholic acid (*m*/*z*: 391.2858 [M – H^–^]). The annotated chemicals in the upregulated area include an exogenous contaminant (1,4-cyclohexanedicarboxylic acid, *m*/*z*: 173.0783 [M + H^+^]) and other seven compounds identified as natural products, drugs, and endogenous metabolites (Figure S10a). For those compounds in the downregulated area, they included endogenous metabolites, exogenous contaminants, drugs and personal care products (Figure S10a).

In urine, we did not observe distinct chemical enrichment between preterm and term birth samples (Figure 2b), despite significant differences in PC1 loadings (*p* < 0.0001). Among the 9225 significantly different LC-MS features between preterm and term birth samples (*p* < 0.05), 19 out of 427 features were tentatively annotated and they were all situated in the downregulated area (Spreadsheet S4). Some of these features annotated were shown as in the volcano plot, e.g., didecyldimethylammonium (*m*/*z*: 326.3782 [M + H]^+^) and adebosine (*m*/*z*: 268.1002 [M + H]^+^) (Figure 3b). The largest number of annotated chemicals belonged to endogenous metabolites and exogenous contaminants (Figure S10b). Only one feature (unknown, *m*/*z*: 704.5230 [M + H]^+^) was present in the upregulated area.

#### 3.4.2 Difference between Urine and Serum

The mean log abundances of chemical features from urine and serum samples showed a positive correlation (*R*^2^ > 0.5), with some chemical features diverging from the regression line before imputation and batch correction for the initial data set (Figure S11a), after imputation and batch correction for the initial data set (Figure S11b), and after imputation and batch correction for the chemical features that have a frequency >70% (Figure S11c). A significant difference in most chemicals was observed between urine and serum samples after batch correction (Figure S12), with two distinct clusters separated with a *p*-value <0.0001 for PC1 between urine and serum samples.

From the volcano plot of 25,885 chemical features (serum versus urine, *p* < 0.05), chemicals were more predominant in serum (3369 chemicals in the upregulated area versus. 739 chemicals in the downregulated area) (Figure S13a). The chemicals with the largest fold change in the downregulated and upregulated areas were tentatively annotated as docosahexaenoic acid (*m*/*z*: 327.2333 [M – H]^−^) and 4-ethoxy ethylbenzoate (*m*/*z*: 195.1018 [M + H]^+^). In the upregulated and downregulated areas, 109 and 20 chemicals, respectively, were tentatively annotated (Spreadsheet S5), with endogenous metabolites and exogenous contaminants being the most frequently annotated (Figure S10c).

#### 3.4.3 Association among Different Chemicals

Twelve significant associations (absolute Pearson *R* > 0.5) in all samples were found between endogenous metabolites and exogenous contaminants (Spreadsheet S4), which only was observed in serum samples. For example, *p*-cresyl sulfate positively correlated with 4-(hydroxymethyl)benzenesulfonic acid and 4-phenol sulfonic acid (Figure S14).

**Figure 4:**
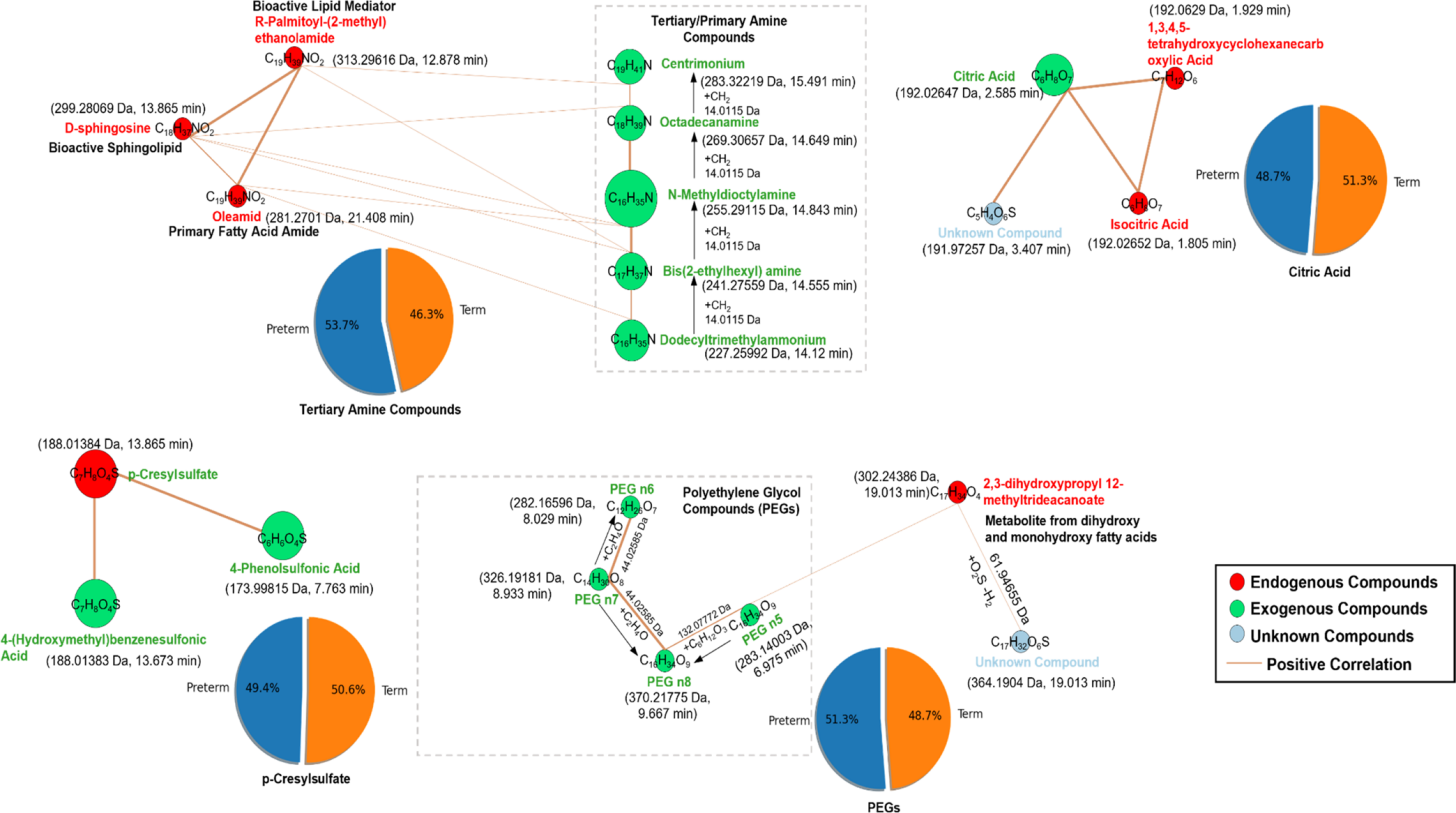
Molecular interaction networks for endogenous (red) and exogenous compounds’ features (green) in serum samples (*N* = 95). The network indicates that the features of MSn had a score of 50, a coverage value of 70, and a minimum number of fragments of 3. The correlation in the networks had *R* values >0.5. The correlations shown in the network are all positive (brown line). The thickness of the line indicates the strength of the correlation. The red circle and green circle represent the endogenous and exogenous compounds, respectively. The blue circle represents the unknown chemicals. The size of the circle indicates the size of the integrated area of the chemical feature. Endogenous and exogenous compounds belong to Level 2 and unknown compounds belong to Level 3 based on Schymanski, Jeon, Gulde, Fenner, Ruff, Singer, and Hollender^21^ for the annotation confidence. The pie charts show the average percentages of preterm and term births associated with exogenous compounds in all serum samples where these compounds were detected, such as the average percentage of poly(ethylene glycol) compounds (PEGs).

The molecular network for significant associations (*R*^2^ > 0.5) between endogenous metabolites and exogenous chemicals were shown in Figure 4. Endogenous-exogenous compound correlations included d-sphingosine with *N*-methyldioctylamine, octadecanamine, and bis(2-ethylhexy)amine. The amine compounds like octadecanamine (primary amine) and bis(2-ethylhexyl) amine (tertiary amine) showed significant associations. Other correlations involved *R*-palmitoyl-(2-methyl) ethanolamide and centrimonium, and oleamide with bis(2-ethylhexyl) amine and bis(2-ethylhexyl) amine and dodecyltrimethylammonium, slightly more occurring in preterm birth (∼53%). PEG n5 was positively correlated with an endogenous metabolite, 2,3-dihydroxypropyl 12-methyltridecanoate. Citric acid was positively correlated with two endogenous metabolites, isocitric acid and 1,3,4,5-tetrahydroxycyclohexanecarboxylic acid.

## 4 Discussion

Among the confirmed compounds, two chemicals, dodecyltrimethylammonium and *N*,*N*-dimethyldecylamine *N*-oxide, widely used in PCPs and as surfactants for various industrial products, appear to not have been previously reported in human samples, based on our searches with BED and HMD. In addition, we found that the azole fungicide propiconazole, a heavily used agricultural agent with carcinogenic^25^ and endocrine-disrupting effects on humans.^26^ Three tertiary amine compounds (triisopropanolamine, tributylamine, diphenylamine) are used in numerous industrial applications such as surfactants and stabilizers, with diphenylamine and its derivatives listed as propriety pollutants by the European Union.^27^ Two phosphate ester flame retardants, tributyl phosphate and triisobutyl phosphate (They were not distinctly annotated due to different RTs but the same MS/MS spectra), were found to have higher detection rates and average concentrations in serum samples compared to paired urine samples (semiquantification shown in Figure S15). This is similar to previous reports where tributyl phosphate was the predominant substance in blood samples from Beijing^28^ and Shenzhen,^29^ China. However, triisobutyl phosphate has not been reported in human samples. 2,2,6,6-Tetramethyl-4-piperidinol, found in PCPs such as cosmetics, was detected in human blood.^30^ 4-Nitrophenol, a metabolite of the organophosphate pesticide methyl parathion, which is illegally applied to the interiors of homes in the US,^31^ it was also detected in our samples. For aminophenols, 2-aminophenol and 3-aminophenol could not be differentiated based on RTs (difference <0.05 min) and were confirmed by product ions (Figure S6b). Aminophenols and their derivatives are commercially important in dyes, petroleum additives, and pharmaceutical industries. Interestingly, the commonly used 4-aminophenol was not detected in our samples, while 2- and 3-aminophenols, which we did detect, are less frequently reported in human samples. All pairs of samples found both 2-aminophenol and 3-aminophenol with good correlation between urine and serum (*R*^2^ = 0.988), suggesting that products exposing pregnant women might contain both aminophenols. We also found that 39 out of the 327 chemicals were not included in BED (Spreadsheet S7).^30^ Among these chemicals, except for dodecyltrimethylammonium (Level 1), citroflex (Level 2) was annotated as exogenous contaminants and PCPs but it is not included in HMD and BED. According to the blood paper count from BED (Spreadsheet S8), several compounds showed a very limited number of studies: dodecyltrimethylammonium (0), *N*,*N*-dimethyldecylamine *N*-oxide, triisopropanolamine (1), and tributylamine (3). Additionally, we identified 11 tentatively annotated compounds with similarly limited study numbers (Spreadsheet S8). These compounds require further investigation to determine their presence in the human body.

Based on the chemical profiles of the samples, we were able to distinguish between preterm birth and term birth in only serum (Figure 2a). Preterm birth is a medical condition with a complex pathogenesis.^32^ Previous reports have shown potential associations of environmental contaminants with preterm birth compared with the control samples, e.g., the pesticide DDT (dichlorodiphenyltrichloroethane),^33^ lead,^34^ and phthalates.^35−37^ For phthalates, diheptyl phthalate (Level 2) was found in preterm birth samples. This is not surprising since phthalate esters are widely used in the plasticizer industry and have been detected in human samples from adults and children in Asia and North America.^38^ While previous studies have reported significant associations of phthalates and their metabolites with the gestational age in other New York City pregnancy cohorts,^39,40^ we were unable to find any associations of chemical features between preterm birth and term birth in either blood or urine samples. Due to the limited sample numbers, we do not further elucidate this observation. It should be noted that phthalates are ubiquitous and can leach from medical supplies^41^ and laboratory equipment,^42^ as seen in our current raw data set where many phthalates were present in laboratory controls and even in solvent blanks, complicating source identification. Therefore, we do not further speculate on the sources of diheptyl phthalate from our samples.

In serum, among all annotated chemicals with features significantly different (*p* < 0.05) in preterm birth samples and 1.2-fold higher abundances compared to term birth samples (Figure 3a), only 1,4-cyclohexanedicarboxylic acid was categorized as an exogenous contaminant. This compound is used in the production of nylon and polyester resins for various purposes, such as enhancing plasticizing efficiency and hardness.^43^ We speculate that products containing this compound may be absorbed by the human body through ingestion and inhalation. Although 1,4-cyclohexanedicarboxylic acid is currently under the TSCA, it is not listed in the BED. To our knowledge, no studies have reported the detection of 1,4-cyclohexanedicarboxylic acid in human samples. Other compounds, such as *p*-coumaric acid, ellagic acid, and bisoprolol, are commonly used in drugs or health products for dietary antioxidants, antioxidant activity, and hypertension management. Regarding endogenous metabolites, deoxycholic acid, a bile acid, is one of the main bile acids present in the meconium of preterm infants, entering the fetus through placental transfer. More recent studies have also shown that changes in total bile acids are directly related to preterm birth rates.^44,45^

For the annotated chemicals that were significantly different (*p* < 0.05) in preterm birth samples, with lower abundances compared to term birth samples, we identified two exogenous contaminants in serum and six in urine. However, these contaminants were not detected with higher frequency in preterm birth samples or in either urine or serum. The negative fold change in these chemicals might be attributed to individual sample variations compared to endogenous metabolites and differences in sampling times for urine.

We also observed that adenosine (an endogenous metabolite), which was significantly different in preterm birth, showed decreased abundances in both serum and urine samples (Figure S13b). Adenosine is a common endogenous nucleoside that generally counteracts ATP-induced effects, such as inflammation.^46^ It has been demonstrated that adenosine levels can increase during normal pregnancy due to platelet activation and elevated nucleosidase activity.^47^ Interestingly, adenosine, a marker of oxidative stress, has been found to be significantly higher in pregnant women with preeclampsia compared to those without the condition.^48^ Lower levels of adenosine in both urine and serum might be linked to preterm birth outcomes. Although endogenous metabolites were not the primary focus of this study, the levels of adenosine associated with preterm birth have not been reported. This warrants further attention from researchers, especially since adenosine is also used as a drug for treating supraventricular tachycardia during pregnancy.^49^ Generally, we observed a broader range of chemicals, both endogenous and exogenous, in serum samples (Figure S13a). This allows for the identification of both biomarker chemicals and exogenous contaminants. Nonetheless, some exogenous contaminants, such as centrimonium, were found to be more enriched in urine samples.

We found that paired prenatal urine and serum samples have different enrichment of chemical features (Figure S12), despite some endogenous chemicals showing a significantly higher proportion in the serum samples (Spreadsheet S2). Of the tentatively identified compounds we detected (Level 2, Spreadsheet S2), many were endogenous compounds or pharmaceuticals and their transformation products as part of metabolism in the human body.

Some endogenous chemicals showed an association with exogenous contaminants in serum. For example, *p*-cresyl sulfate (*p*-CS) correlated with 4-phenolsulfonic acid (4-PSA) and 4-(hydroxymethyl)benzenesulfonic acid (4-HMBSA) (Figure 4). *p*-CS is a prototype protein-bound molecule derived from the secondary metabolism of *p*-cresol, where increased concentrations can be associated with deteriorating kidney function.^50^ 4-PSA is a common intermediate/component of surfactants, detergents, pharmaceuticals, and dyes. 4-HMBSA is a derivative of substituted benzenesulfonic acids, widely used as intermediates for organic compound synthesis. 4-PSA has been listed in the ToxCast database,^51^ while the human toxicity for both 4-PSA and 4-HMBSA is not clear. In the current network, significant relationships were observed among PEGs, composed of polyether compounds with repeating ethylene glycol units. PEGs are used as components in drugs and PCPs. Narrowly defined molecular weight ranges of PEGs are often produced as a commercial mixture,^52^ similar to our data showing a correlated pattern with the loss of ethylene oxide (C_2_H_4_O, 44.02585 Da) among PEGs n5–8. PEG n5 was observed to have a positive connection to 2,3-dihydroxypropyl 12-methyltridecanoate, an endogenous metabolite from the 12-methyltridecanoate fatty acid chain, and a complex microbial-related metabolite in gastric cancer.^53^ Only high-molecular-weight PEGs (>400 Da, e.g., PEG n8) have shown toxic effects in animals,^54^ and we were not able to find any toxicity studies on these PEGs. Another interesting correlation was observed between a group of tertiary amine compounds, used as chemical intermediates/surfactants, with a mass defect of −CH_2_– group (14.0115 Da), e.g., centrimonium and octadecanamine, and fatty acid amide (oleamide) and bioactive lipid metabolites (d-sphingosine and *R*-palmitoyl-(2-methyl) ethanolamide) (Figure 4). This suggests that these amine compounds might interfere with lipid and fatty acid metabolism. This can be referenced by a relevant report indicating that surfactants solubilize lipid membranes and transform them into lipid-surfactant micelles, while fatty acids transform lipids into cubic and hexagonal phases.^55^ All these associations indicate the potential direct or indirect intervention of exogenous contaminants on the metabolism processes in human bodies.

In our data set, most annotated chemical features could not be fully confirmed due to the lack of analytical standards. The endogenous metabolites and exogenous contaminants groups had significantly more compounds in them than drugs, natural products and personal care products (Figure S3). Given the abundance of environmental contaminants and their observed associations with endogenous metabolites, many of these contaminants could substantially contribute to the exposome^56^ disturb metabolic pathways such as lipid metabolism and inflammation regulation.^57^

## 5 Limitations and Recommendations

While our study presents some evidence associating chemical exposures with preterm birth, our study is not a comprehensive epidemiological study, but a human exposure study. Our main goal was to identify exogenous contaminants that have not been detected in human samples and to highlight them for further studies regarding their potentially adverse effects on humans, such as preterm birth in pregnant women. We have six limitations in our study that need to be acknowledged:

(1) We were limited to only 95 participants with paired urine and serum samples (including 35 pairs from preterm births). Despite the limited data set, it does not impact the annotation workflow. It is recommended to have a larger sample size to establish strong associations between contaminants and endogenous metabolites in pregnant women with preterm births, and to explore which groups of pregnant women have higher or lower detection frequencies of exogenous contaminants.
(2) Although we observed clustering in the serum heatmap at a chemical detection frequency cutoff of 70% (as well as at 60 and 80%, as shown in Figures 2a and S16) between preterm and term births, we did not observe a similar pattern of chemical enrichment in the paired urine samples across detection frequencies of 60–80% (Figures 3b and S17). This discrepancy may be due to the different sampling times for urine and the more pronounced matrix effects in urine.
(3) The RTs of the QC compounds (1.5–23.1 min) in the LC-MS run generally spanned most of the total run time (0–25 min). However, the 51 QC compounds were not able to represent all compounds eluted from the LC columns during non-targeted analysis due to the varying physicochemical properties of the numerous compounds present in complex samples, especially for early eluting polar molecules. These QC compounds were used solely to assess instrument stability in each batch run. We also lacked internal standards to improve the accuracy of semiquantification, which requires further investigation to enhance.
(4) Approximately ∼22% of features (Level ≥3) were tentatively annotated by matching to spectral databases, and 12 chemicals were confirmed by authentic standards. These annotated chemicals also exhibit significant data gaps when compared to the differentially abundant chemical features between preterm and term births. We recommend further development of additional suspect or non-targeted screening methods to identify the chemical features that show significant differences between the groups.
(5) The analytical instrument presents challenges related to varying setting parameters across different mass spectrometers and manufacturers, especially for soft ionization techniques. In non-targeted analysis (NTA), the desired mass resolving power may not be achieved for specific masses. For Orbitrap HRMS in NTA, the upper limit of mass resolving power can lead to ion loss and dephasing of oscillations. The limited number of ions per unit time entering the C-trap (AGC targets) could significantly affect the sensitivity for small molecule chemicals with lower detection frequencies in our study. We recommend multiple scans of different mass ranges for pooled samples with a dynamic MS^2^ data window to mitigate the limited AGC targets per scan. Additionally, it is advisable to combine various analytical approaches to expand chemical space coverage, such as using GC separation for volatile and highly nonpolar chemicals in conjunction with Quadrupole Time-of-Flight (QTOF) MS.
(6) Considering the broader spectrum of compounds’ positive and negative ionization abilities, further studies should include a second injection using neutral or basic mobile phase conditions to extend chemical coverage.

## Supporting Information Available

Individual compounds information (e.g., annotation and detection frequency) and statistical results (e.g., fold-change and *p*-values) (XLSX)
Workflow, data normalization, detailed instrumental parameters, and examples of compounds confirmed using analytical standards, as well as specifically analyzed data (PDF)

## References

[ref1] Muir D. C. G.; Getzinger G. J.; McBride M.; Ferguson P. L. How Many Chemicals in Commerce Have Been Analyzed in Environmental Media? A 50 Year Bibliometric Analysis. Environ. Sci. Technol. 2023, 57 (25), 9119–9129. 10.1021/acs.est.2c09353. 37319372 PMC10308830

[ref2] Yang D.; Liu Q.; Wang S.; Bozorg M.; Liu J.; Nair P.; Balaguer P.; Song D.; Krause H.; Ouazia B.; Abbatt J. P. D.; Peng H. Widespread formation of toxic nitrated bisphenols indoors by heterogeneous reactions with HONO. Sci. Adv. 2022, 8 (48), eabq7023 10.1126/sciadv.abq7023. 36459560 PMC10936053

[ref3] Tian Z.; Zhao H.; Peter K. T.; Gonzalez M.; Wetzel J.; Wu C.; Hu X.; Prat J.; Mudrock E.; Hettinger R.; Cortina A. E.; Biswas R. G.; Kock F. V. C.; Soong R.; Jenne A.; Du B.; Hou F.; He H.; Lundeen R.; Gilbreath A.; Sutton R.; Scholz N. L.; Davis J. W.; Dodd M. C.; Simpson A.; McIntyre J. K.; Kolodziej E. P. A ubiquitous tire rubber–derived chemical induces acute mortality in coho salmon. Science 2021, 371 (6525), 185–189. 10.1126/science.abd6951. 33273063

[ref4] Wang Z.; Walker G. W.; Muir D. C. G.; Nagatani-Yoshida K. Toward a Global Understanding of Chemical Pollution: A First Comprehensive Analysis of National and Regional Chemical Inventories. Environ. Sci. Technol. 2020, 54 (5), 2575–2584. 10.1021/acs.est.9b06379. 31968937

[ref5] Rappaport S. M. Implications of the exposome for exposure science. J. Exposure Sci. Environ. Epidemiol. 2011, 21 (1), 5–9. 10.1038/jes.2010.50. 21081972

[ref6] Wang Y.; Hollis-Hansen K.; Ren X.; Qiu Y.; Qu W. Do environmental pollutants increase obesity risk in humans?. Obes. Rev. 2016, 17 (12), 1179–1197. 10.1111/obr.12463. 27706898

[ref7] Chatkin J.; Correa L.; Santos U. External Environmental Pollution as a Risk Factor for Asthma. Clin. Rev. Allergy Immunol. 2022, 62 (1), 72–89. 10.1007/s12016-020-08830-5. 33433826 PMC7801569

[ref8] Tuovinen S.; Eriksson J. G.; Kajantie E.; Räikkönen K. Maternal hypertensive pregnancy disorders and cognitive functioning of the offspring: a systematic review. J. Am. Soc. Hypertens. 2014, 8 (11), 832–847.e1. 10.1016/j.jash.2014.09.005. 25455009

[ref9] Nobles C. J.; Grantz K. L.; Liu D.; Williams A.; Ouidir M.; Seeni I.; Sherman S.; Mendola P. Ambient air pollution and fetal growth restriction: Physician diagnosis of fetal growth restriction versus population-based small-for-gestational age. Sci. Total Environ. 2019, 650, 2641–2647. 10.1016/j.scitotenv.2018.09.362. 30296771 PMC6203640

[ref10] Tau G. Z.; Peterson B. S. Normal Development of Brain Circuits. Neuropsychopharmacology 2010, 35 (1), 147–168. 10.1038/npp.2009.115. 19794405 PMC3055433

[ref11] Stiles J.; Jernigan T. L. The Basics of Brain Development. Neuropsychol. Rev. 2010, 20 (4), 327–348. 10.1007/s11065-010-9148-4. 21042938 PMC2989000

[ref12] LaKind J. S.; Goodman M.; Naiman D. Q. Use of NHANES Data to Link Chemical Exposures to Chronic Diseases: A Cautionary Tale. PLoS One 2012, 7 (12), e51086 10.1371/journal.pone.0051086. 23227235 PMC3515548

[ref13] Vermeulen R.; Schymanski E. L.; Barabási A.-L.; Miller G. W. The exposome and health: Where chemistry meets biology. Science 2020, 367 (6476), 392–396. 10.1126/science.aay3164. 31974245 PMC7227413

[ref14] Caballero-Casero N.; Belova L.; Vervliet P.; Antignac J.-P.; Castaño A.; Debrauwer L.; López M. E.; Huber C.; Klanova J.; Krauss M.; Lommen A.; Mol H. G. J.; Oberacher H.; Pardo O.; Price E. J.; Reinstadler V.; Vitale C. M.; van Nuijs A. L. N.; Covaci A. Towards harmonised criteria in quality assurance and quality control of suspect and non-target LC-HRMS analytical workflows for screening of emerging contaminants in human biomonitoring. TrAC, Trends Anal. Chem. 2021, 136, 116201 10.1016/j.trac.2021.116201.

[ref15] López A.; Dualde P.; Yusà V.; Coscollà C. Retrospective analysis of pesticide metabolites in urine using liquid chromatography coupled to high-resolution mass spectrometry. Talanta 2016, 160, 547–555. 10.1016/j.talanta.2016.07.065. 27591649

[ref16] Musatadi M.; Caballero C.; Mijangos L.; Prieto A.; Olivares M.; Zuloaga O. From target analysis to suspect and non-target screening of endocrine-disrupting compounds in human urine. Anal. Bioanal. Chem. 2022, 414 (23), 6855–6869. 10.1007/s00216-022-04250-w. 35904524 PMC9436830

[ref17] Abrahamsson D. P.; Wang A.; Jiang T.; Wang M.; Siddharth A.; Morello-Frosch R.; Park J.-S.; Sirota M.; Woodruff T. J. A Comprehensive Non-targeted Analysis Study of the Prenatal Exposome. Environ. Sci. Technol. 2021, 55 (15), 10542–10557. 10.1021/acs.est.1c01010. 34260856 PMC8338910

[ref18] Manz K. E.; Feerick A.; Braun J. M.; Feng Y.-L.; Hall A.; Koelmel J.; Manzano C.; Newton S. R.; Pennell K. D.; Place B. J.; Pollitt K. J. G.; Prasse C.; Young J. A. Non-targeted analysis (NTA) and suspect screening analysis (SSA): a review of examining the chemical exposome. J. Exposure Sci. Environ. Epidemiol. 2023, 33 (4), 524–536. 10.1038/s41370-023-00574-6. PMC10403360 37380877

[ref19] Moschet C.; Anumol T.; Lew B. M.; Bennett D. H.; Young T. M. Household Dust as a Repository of Chemical Accumulation: New Insights from a Comprehensive High-Resolution Mass Spectrometric Study. Environ. Sci. Technol. 2018, 52 (5), 2878–2887. 10.1021/acs.est.7b05767. 29437387 PMC7239036

[ref20] Sobus J. R.; Grossman J. N.; Chao A.; Singh R.; Williams A. J.; Grulke C. M.; Richard A. M.; Newton S. R.; McEachran A. D.; Ulrich E. M. Using prepared mixtures of ToxCast chemicals to evaluate non-targeted analysis (NTA) method performance. Anal. Bioanal. Chem. 2019, 411 (4), 835–851. 10.1007/s00216-018-1526-4. 30612177 PMC6469933

[ref21] Schymanski E. L.; Jeon J.; Gulde R.; Fenner K.; Ruff M.; Singer H. P.; Hollender J. Identifying Small Molecules via High Resolution Mass Spectrometry: Communicating Confidence. Environ. Sci. Technol. 2014, 48 (4), 2097–2098. 10.1021/es5002105. 24476540

[ref22] Johnson W. E.; Li C.; Rabinovic A. Adjusting batch effects in microarray expression data using empirical Bayes methods. Biostatistics 2007, 8 (1), 118–127. 10.1093/biostatistics/kxj037. 16632515

[ref23] Waskom M. seaborn: statistical data visualization. J. Open Source Software 2021, 6 (60), 3021 10.21105/joss.03021.

[ref24] Phillips K. A.; Chao A.; Church R. L.; Favela K.; Garantziotis S.; Isaacs K. K.; Meyer B.; Rice A.; Sayre R.; Wetmore B. A.; Yau A.; Wambaugh J. F. Suspect Screening Analysis of Pooled Human Serum Samples Using GC × GC/TOF-MS. Environ. Sci. Technol. 2024, 58 (4), 1802–1812. 10.1021/acs.est.3c05092. 38217501 PMC11459241

[ref25] Edwards D. Reregistration Eligibility Decision (RED) for Permethrin; United States Environmental Protection Agency, 2006.

[ref26] Taxvig C.; Vinggaard A. M.; Hass U.; Axelstad M.; Metzdorff S.; Nellemann C. Endocrine-disrupting properties in vivo of widely used azole fungicides. Int. J. Androl. 2008, 31 (2), 170–177. 10.1111/j.1365-2605.2007.00838.x. 18067565

[ref27] Drzyzga O. Diphenylamine and derivatives in the environment: a review. Chemosphere 2003, 53 (8), 809–818. 10.1016/S0045-6535(03)00613-1. 14505701

[ref28] Zhao F.; Chen M.; Gao F.; Shen H.; Hu J. Organophosphorus Flame Retardants in Pregnant Women and Their Transfer to Chorionic Villi. Environ. Sci. Technol. 2017, 51 (11), 6489–6497. 10.1021/acs.est.7b01122. 28516762

[ref29] Zhao F.; Wan Y.; Zhao H.; Hu W.; Mu D.; Webster T. F.; Hu J. Levels of Blood Organophosphorus Flame Retardants and Association with Changes in Human Sphingolipid Homeostasis. Environ. Sci. Technol. 2016, 50 (16), 8896–8903. 10.1021/acs.est.6b02474. 27434659

[ref30] Barupal D. K.; Fiehn O. Generating the Blood Exposome Database Using a Comprehensive Text Mining and Database Fusion Approach. Environ. Health Perspect. 2019, 127 (9), 097008 10.1289/EHP4713. 31557052 PMC6794490

[ref31] Hryhorczuk D. O.; Moomey M.; Burton A.; Runkle K.; Chen E.; Saxer T.; Slightom J.; Dimos J.; McCann K.; Barr D. Urinary p-nitrophenol as a biomarker of household exposure to methyl parathion. Environ. Health Perspect. 2002, 110 (suppl 6), 1041–1046. 10.1289/ehp.02110s61041. PMC1241290 12634137

[ref32] Goldenberg R. L.; Culhane J. F.; Iams J. D.; Romero R. Epidemiology and causes of preterm birth. Lancet 2008, 371 (9606), 75–84. 10.1016/S0140-6736(08)60074-4. 18177778 PMC7134569

[ref33] Longnecker M. P.; Klebanoff M. A.; Zhou H.; Brock J. W. Association between maternal serum concentration of the DDT metabolite DDE and preterm and small-for-gestational-age babies at birth. Lancet 2001, 358 (9276), 110–114. 10.1016/S0140-6736(01)05329-6. 11463412

[ref34] Andrews K. W.; Savitz D. A.; Hertz-Picciotto I. Prenatal lead exposure in relation to gestational age and birth weight: A review of epidemiologic studies. Am. J. Ind. Med. 1994, 26 (1), 13–32. 10.1002/ajim.4700260103. 8074121

[ref35] Meeker J. D.; Hu H.; David E. C.; Lamadrid-Figueroa H.; Antonia M. C.; Adrienne S. E.; Hernandez-Avila M.; Loch-Caruso R.; Martha M. T.-R. Urinary Phthalate Metabolites in Relation to Preterm Birth in Mexico City. Environ. Health Perspect. 2009, 117 (10), 1587–1592. 10.1289/ehp.0800522. 20019910 PMC2790514

[ref36] Hauser R.; Calafat A. M. Phthalates and human health. Occup. Environ. Med. 2005, 62 (11), 806–818. 10.1136/oem.2004.017590. 16234408 PMC1740925

[ref37] Silva M. J.; Samandar E.; Preau J. L.; Reidy J. A.; Needham L. L.; Calafat A. M. Quantification of 22 phthalate metabolites in human urine. J. Chromatogr. B 2007, 860 (1), 106–112. 10.1016/j.jchromb.2007.10.023. 17997365

[ref38] Domínguez-Romero E.; Komprdová K.; Kalina J.; Bessems J.; Karakitsios S.; Sarigiannis D. A.; Scheringer M. Time-trends in human urinary concentrations of phthalates and substitutes DEHT and DINCH in Asian and North American countries (2009–2019). J. Exposure Sci. Environ. Epidemiol. 2023, 33 (2), 244–254. 10.1038/s41370-022-00441-w. PMC10005949 35513587

[ref39] Wolff M. S.; Stephanie M. E.; Gertrud S. B.; Ye X.; Manori J. S.; Zhu C.; Wetmur J.; Antonia M. C. Prenatal Phenol and Phthalate Exposures and Birth Outcomes. Environ. Health Perspect. 2008, 116 (8), 1092–1097. 10.1289/ehp.11007. 18709157 PMC2516577

[ref40] Whyatt R.; Adibi J.; Calafat A.; Rundle A.; Just A.; Hauser R. Maternal prenatal urinary concentrations of di-(2-ethylhexyl) phthalate in relation to the timing of labor: results from a birth cohort study of inner-city mothers and newborns. Epidemiology 2008, 19 (6), S220.

[ref41] Wang W.; Kannan K. Leaching of Phthalates from Medical Supplies and Their Implications for Exposure. Environ. Sci. Technol. 2023, 57 (20), 7675–7683. 10.1021/acs.est.2c09182. 37154399 PMC10210534

[ref42] Oca M. L.; Rubio L.; Sarabia L. A.; Ortiz M. C. Dealing with the ubiquity of phthalates in the laboratory when determining plasticizers by gas chromatography/mass spectrometry and PARAFAC. J. Chromatogr. A 2016, 1464, 124–140. 10.1016/j.chroma.2016.07.074. 27507728

[ref43] Yuhong C.; xi L.; Maosheng Z. Synthesis of Poly(1,4-cyclohexanedimethyl-1,4-cyclohexanedicarboxylate) as the Matrix Resin for Transparent Composites. Polym. Polymer Compos. 2011, 19 (2–3), 123–130. 10.1177/0967391111019002-312.

[ref44] You S.; Cui A.-M.; Hashmi S. F.; Zhang X.; Nadolny C.; Chen Y.; Chen Q.; Bush X.; Hurd Z.; Ali W.; Qin G.; Deng R. Dysregulation of bile acids increases the risk for preterm birth in pregnant women. Nat. Commun. 2020, 11 (1), 2111 10.1038/s41467-020-15923-4. 32355283 PMC7193585

[ref45] Ma Z.; Liu Y.; Chai L.; Jin G.; Sun Y.; Zhou S.; Yin P.; Wang S.; Zhu Y.; Zhang D.; Lu S.; Zhu B. Metabolic changes in bile acids with pregnancy progression and their correlation with perinatal complications in intrahepatic cholestasis of pregnant patients. Sci. Rep. 2023, 13 (1), 1608 10.1038/s41598-022-22974-8. 36709211 PMC9884190

[ref46] Bours M. J. L.; Swennen E. L. R.; Di Virgilio F.; Cronstein B. N.; Dagnelie P. C. Adenosine 5′-triphosphate and adenosine as endogenous signaling molecules in immunity and inflammation. Pharmacol. Ther. 2006, 112 (2), 358–404. 10.1016/j.pharmthera.2005.04.013. 16784779

[ref47] Spaans F.; de Vos P.; Bakker W. W.; van Goor H.; Faas M. M. Danger Signals From ATP and Adenosine in Pregnancy and Preeclampsia. Hypertension 2014, 63 (6), 1154–1160. 10.1161/HYPERTENSIONAHA.114.03240. 24688119

[ref48] Espinoza J.; Espinoza A. F.; Power G. G. High fetal plasma adenosine concentration: a role for the fetus in preeclampsia?. Am. J. Obstet. Gynecol. 2011, 205 (5), 485.e24–485.e27. 10.1016/j.ajog.2011.06.034. 21855848

[ref49] Ibetoh C. N.; Stratulat E.; Liu F.; Wuni G. Y.; Bahuva R.; Shafiq M. A.; Gattas B. S.; Gordon D. K. Supraventricular Tachycardia in Pregnancy: Gestational and Labor Differences in Treatment. Cureus 2021, 13 (10), e18479 10.7759/cureus.18479. 34659918 PMC8494174

[ref50] Duranton F.; Cohen G.; De Smet R.; Rodriguez M.; Jankowski J.; Vanholder R.; Argiles A. Normal and Pathologic Concentrations of Uremic Toxins. J. Am. Soc. Nephrol. 2012, 23 (7), 1258–1270. 10.1681/ASN.2011121175. 22626821 PMC3380651

[ref51] U. E. O. ToxCast Database: Invitrodb, version 4.1; Center for Computational Toxicology, 2023.

[ref52] Jang H.-J.; Shin C. Y.; Kim K.-B. Safety Evaluation of Polyethylene Glycol (PEG) Compounds for Cosmetic Use. Toxicol. Res. 2015, 31 (2), 105–136. 10.5487/TR.2015.31.2.105. 26191379 PMC4505343

[ref53] Yang Y.; Dai D.; Jin W.; Huang Y.; Zhang Y.; Chen Y.; Wang W.; Lin W.; Chen X.; Zhang J.; Wang H.; Zhang H.; Teng L. Microbiota and metabolites alterations in proximal and distal gastric cancer patients. J. Transl. Med. 2022, 20 (1), 439 10.1186/s12967-022-03650-x. 36180919 PMC9524040

[ref54] Fang J.-L.; Vanlandingham M. M.; Beland F. A.; Felton R. P.; Maisha M. P.; Olson G. R.; Patton R. E.; Rosenberg A. S.; da Costa G. G. Toxicity of high-molecular-weight polyethylene glycols in Sprague Dawley rats. Toxicol. Lett. 2022, 359, 22–30. 10.1016/j.toxlet.2022.01.011. 35092809 PMC8932377

[ref55] Lichtenberg D.; Ahyayauch H.; Goñi F. M. The Mechanism of Detergent Solubilization of Lipid Bilayers. Biophys. J. 2013, 105 (2), 289–299. 10.1016/j.bpj.2013.06.007. 23870250 PMC3714928

[ref56] Rietjens I. M. C. M.; Michael A.; Bolt H. M.; Siméon B.; Andrea H.; Nils H.; Christine K.; Angela M.; Gloria P.; Daniel R.; Natalie T.; Gerhard E. The role of endogenous versus exogenous sources in the exposome of putative genotoxins and consequences for risk assessment. Arch. Toxicol. 2022, 96 (5), 1297–1352. 10.1007/s00204-022-03242-0. 35249149 PMC9013691

[ref57] Ross M. K.; Matthews A. T.; Mangum L. C. Chemical Atherogenesis: Role of Endogenous and Exogenous Poisons in Disease Development. Toxics 2014, 2, 17–34. 10.3390/toxics2010017. 25705646 PMC4333738

