# Supplementary material for: Insights into the Chemical Exposome during Pregnancy: A Non-Targeted Analysis of Preterm and Term Births
Source: Environ Sci Technol. 2024 Nov 11;58(47):20883–93. doi: 10.1021/acs.est.4c08534 (PMC11603774; doi:10.1021/acs.est.4c08534)
Supplement: Supplementary file 2 — es4c08534_si_002.pdf [file es4c08534_si_002.pdf]

Supporting Information

Insights into the Chemical Exposome during Pregnancy: A Non-Targeted Analysis of Preterm and Term Births

Xiaowen Ji[1], Mathusa Lakuleswaran[1], Whitney Cowell[1], Linda G. Kahn[1], Marina Sirota[2,4] and Dimitri Abrahamsson[1,3*]

[1] *Division of Environmental Pediatrics, Department of Pediatrics, Grossman School of Medicine, New York University, New York, NY 10016, the United States of America*

[2] *Bakar Computational Health Sciences Institute, UCSF, San Francisco, CA 94158, the United States of America*

[3] *Department of Obstetrics, Gynecology and Reproductive Sciences, University of California, San Francisco, San Francisco, California 94158, United States of America*

[4] *Department of Pediatrics, University of California, San Francisco, San Francisco, California 94158, United States of America*

* Corresponding author's e-mails: jixiaowen4321@qq.com; dimitri.abrahamsson@gmail.com

Summary

Pages: 32

Graphs:18

Texts: 5

Spreadsheets: 8

Contents of Texts, Table, and Figures

Text S1. The creatinine normalization approach to diluted urine samples S7

Text S2. Levels of identification confidence S7

Text S3. The basic parameters for MS-DIAL and Compound Discoverver S8

Text S4. The instrument method S10

Text S5. Quality assurance/quality control (QA/QC) S10

Figure S1. Workflow diagram of sample treatment and chemical analysis, data cleansing and processing, and data analysis for the urine and serum samples collected from 95 pregnant participants in NYU CHES. S12

Figure S2. The highest point or peak of a chromatographic or mass spectrometric signal and abundances (log transformed) of retention times (RT) and molecular mass (g/mol). The plot displays ions from the full scan of the individual samples (**a** and **b**) and the full scan/ddMS^2 of the pooled samples (**c** and **d**) in both positive and negative electrospray ionization modes. S14

Figure S3. Data analysis before and after "Combat" batch correction for the four individual batches in serum (**a**), urine (**b**), and combined urine & serum samples (**c**). The example results demonstrate the following: For preterm and term birth samples in serum or urine (a/b): (**1**) color-labeled principal components analysis (PCA) loadings by preterm and term birth, and (**2**) color-labeled PCA loadings by batch. For combined serum and urine samples (c): (**1**) color-labeled PCA loadings by sample type (serum and urine), and (**2**) color-labeled PCA loadings by batch. S16

Figure S4. The dataset analysis was conducted after processing with the "Combat" batch correction to examine correlations and statistically significant differences among principal component (PC) loadings, batch, and sample type. The batch correction was applied for preterm and term birth samples across four batches in both urine and serum, as well as for combined urine and serum samples in four batches. The Pearson R values and p-values were reported for urine (**a** and **b**), serum (**c** and **d**), and combined serum & urine (**e** and **f**). PT: preterm and term birth sample types, SvU: serum and urine sample types. S18

Figure S5. Level 2 chemicals annotated as endogenous metabolites, exogenous contaminants, drugs, natural products, and personal care products. The bar chart shows the full tally of annotated chemicals in each specific category, plotting with the decreasing number of chemicals. The red and grey balls represent the annotated category and non-annotated category, respectively. S19

Figure S6. Examples of compounds using authentic standards detected by UHPLC-Orbitrap MS with ESI- (**a**) and ESI+ (**b**) polarities. The chromatographic plot of 4-nitrophenol and aminophenol isomers in the authentic standards and real samples. The compounds were initially selected by comparing different databases and were further confirmed by authentic standards. The differential plot of the deconvoluted spectrum between real samples and authentical standards. S20

Figure S7. Clustering heatmap of chemical abundances after batch effect correction for serum (S) and urine samples (U) (**a**) and the boxplot for the significant difference ($p < 0.001$) of PCs 1-3 between serum and urine samples using Mann-Whitney-Wilcoxon test two-sided with Bonferroni correction (**b**). The bottom and top of boxes represent the 25th and 75th percentiles, the error bars denote 10th to 90th percentiles, the solid line means the median value. The total number of chemical features (combing ESI^+ and ESI^-) is 1524 with the annotation levels 1-3 from the classification of Schymanski, et al. [3]. S21

Figure S8. The detection frequency (%) of annotated chemicals (Levels 1 and 2) classified as exogenous contaminants in preterm and term birth samples: chemicals with higher detection frequency in preterm birth only serum (**a**); and in only urine (**b**). The chemical names in red represent the confirmed chemicals (Level 1) by the authentic standards. S22

Figure S9. The heatmap of detection frequency (%) for annotated chemicals (Levels 1 and 2) classified as exogenous contaminants in preterm and term birth in serum and urine samples, respectively. The specific number of frequencies is shown in the spreadsheet S1. .... S23

Figure S10. The bar chart of the number of chemicals that were successfully annotated, located in the down- ($p < 0.05$, $\log_2$fold < -1.2) and up- regulated areas ($p < 0.05$, $\log_2$fold >1.2) of the serum vs. urine samples / preterm and term birth samples in serum or urine from the volcano plot (Figure 3). S24

Figure S11. Linear regression correlation analysis between urine and serum abundances in logarithm scale (n = 190 urine and serum). The results show: (**a**) all original dataset before imputation and batch correction, (**b**) all dataset after imputation and batch correction, and (**c**) the ions filtered by the cut-off frequency of 70% for imputation and batch correction. S25

Figure S12. Clustering heatmap after batch effect correction for serum and urine samples. The chemical features reveal the differential enrichment between serum and urine samples after multiple testing correction (Benjamini-Hochberg test, 5% false discovery rate). For the differential enrichment between serum and urine samples, 26,038 out of 37,270 chemical features exhibited significant differences ($p <$ 0.05). The boxplots show the statistical difference of principal component 1 (PC1) between urine and serum samples using the Mann-Whitney-Wilcoxon test (two-sided) with Bonferroni correction. The bottom and

top of the boxes represent the 25th and 75th percentiles, the error bars denote the 10th to 90th percentiles, and the solid line indicates the median value. S26

Figure S13. The volcano plot of the log-transformed ratios and corresponding p-values of chemical features with a cut-off frequency of 70% from ESI^+ and ESI^- modes illustrates the data: the statistical differences in chemical features between serum and urine (**a**). The horizontal dashed line indicates the cutoff for the log *p*-value ($p < 0.05$), and the vertical dashed lines indicate the cutoff for fold change (Log_2 fold change = 1.2). The arrow graph (**b**) indicates the regulation status of the same annotated chemical across different volcano plots (**Figure 3a&b** and **this figure a**). Up arrows represent up-regulated areas, while down arrows indicate down-regulated areas. Red balls denote annotated categories, and grey balls represent non-annotated categories. DCA: Deoxycholic Acid, 4-EEB: 4-Ethoxy ethylbenzoate, 4-HBA: 4-Hydroxybenzaldehyde, MEDHHP: Methyl 2-[4-ethenyl-2,6-dihydroxy-3-(3-hydroxyprop-1-en-2-yl)-4-methylcyclohexyl]prop-2-enoate, DHDDIAD: 1,4-dihydroxy-1,4-dimethyl-7-(propan-2-ylidene)-decahydroazulen-6-one, BC: Benzoic Acid. S27

Figure S14. Correlation between p-cresylfulfate and 4-(hydroxymethyl)benzenesulfonic acid, and p-cresylfulfate and 4-phenolsulfonic acid in logarithm scale (n = 95 serum). S28

Figure S15. The semi-quantification of confirmed compounds (level 1) in urine and serum samples based on the integral peak areas of 500 ng/mL analytical standards. S29

Figure S16. Clustering heatmap after batch effect correction (between preterm and term birth) for serum samples. The chemical features reveal the differential enrichment in preterm versus term births among serum with the cut-off detection frequencies of 60% and 80% after multiple testing correction (Benjamini-Hochberg test, 5% false discovery rate). For the differential enrichment in preterm versus term birth samples, 1,791 out of 43,450 chemical features in a detection frequency cut-off of 60% and 1,214 out of 25,323 in a detection frequency cut-off of 80% showed significant differences ($p < 0.05$). S30

Figure S17. Clustering heatmap after batch effect correction (between preterm and term birth) for urine samples. The chemical features reveal the differential enrichment in preterm versus term births among serum with the cut-off detection frequencies of 60% and 80% after multiple testing correction (Benjamini-Hochberg test, 5% false discovery rate). For the differential enrichment in preterm versus term birth samples, 9,518 out of 49,350 chemical features in a detection frequency cut-off of 60% and 8,398 out of 29,448 in a detection frequency cut-off of 80% showed significant differences ($p < 0.05$). S31

Figure S18. The average m/z values of quality control (QC) compounds at their corresponding average retention times (RT, min) across the LC-MS running time. S32

References S33

Contents of SI Spreadsheets

Spreadsheet S1. Quality control standards, including EPA phthalate esters mixtures and an in-house analytical standards mixtures, were used to assess the stability of the UHPLC-Orbitrap mass spectrometer for each batch. The evaluation included mass accuracy, retention time (RT) shifts, and r-values, all derived from the average results across the four batches.

Spreadsheet S2. 345 annotated chemical features, ranging from Level 1 (confirmed by authentic standards) to Level 2 (tentatively annotated by MS1/MS2 database matches), were identified in both ESI^+ and ESI^- modes. These features include those with the best match rates, and their frequency in urine/serum samples from preterm and term births.

Spreadsheet S3. The chemical features were confirmed in our laboratory using authentic standards by matching retention time (RT, min), MS^1 (m/z), and MS^2 (m/z).

Spreadsheet S4. The annotated chemicals that showed significant differences ($p < 0.05$) between preterm and term birth samples were identified in the upregulated region (fold change > 1.2) and the downregulated region (fold change < -1.2) of the volcano plot, in either serum or urine samples.

Spreadsheet S5. The annotated chemicals that showed significant differences ($p < 0.05$) between serum and urine samples were identified in the upregulated region (fold change > 1.2) and the downregulated region (fold change < -1.2) of the volcano plot.

Spreadsheet S6. The Pearson correlation (R) matrix was analyzed between exogenous contaminants and endogenous metabolites in serum samples (no correlation was found in urine samples).

Spreadsheet S7. The chemicals (both endogenous and exogenous) were not listed in the Blood Exposome Database (https://bloodexposome.org/).

Spreadsheet S8. The blood paper count for chemicals annotated as exogenous contaminants, which have a higher detection frequency in either serum or urine samples, was obtained from the Blood Exposome Database.

Text S1. The creatinine normalization approach to diluted urine samples

Urine samples can be diluted under various conditions, such as increased water intake or diuretic use. According to the Substance Abuse and Mental Health Services Administration (SAMHSA) guidelines (https://www.federalregister.gov/d/2023-21734), a urine sample is considered diluted if the creatinine concentration is below 20 mg/dL. In our study, we used the creatinine normalization method, as employed in similar research.[1,2] Based on the creatinine concentrations from 95 pregnant women in our study, we established 25.22 mg/dL as the reference concentration for undiluted samples based on the calculation of the average concentration of undiluted urine. For quantification, we employed a six-point calibration curve ranging from 1 to 95 mg/dL, spiked with 50 μg/L of an internal standard (creatinine-d3), using isotope dilution (with linearity > 0.99 for creatinine). Data acquisition and processing were carried out with Xcalibur v. 4.3 (including Freestyle 1.6 and Quan browser). The chemical abundances in diluted urine samples were adjusted using the following equation:

$$\text{Chemical abundance after normalized creatinin} = \text{initial abundance } \times \frac{\text{Reference Creatinine}}{\text{Sample Creatinine}}$$

Text S2. Levels of identification confidence

The various confidence levels are as follows:

Level 1: structure confirmed by a chemical standard with MS/MS and retention time (RT) matching.

Level 2: probable structure deduced by spectrum database matching or other diagnostic evidence (e.g., parent ion information and MS/MS).

Level 3: tentative candidate(s) supported by partial evidence for possible structure(s), but insufficient evidence for the exact structure(s).

Level 4: an unequivocal molecular formula can be assigned through the spectral information but not enough information to propose possible structures.

Level 5: only exact mass (m/z) with insufficient information to assign a formula.

Text S3. The basic parameters for MS-DIAL and Compound Discoverver

MS-DIAL:

MS1 tolerance: 0.0025 Da; MS2 tolerance: 0.0015 Da.

Retention time: 0 – 25 min.

MS1 mass range: 50 – 1000 Da.

MS/MS mass range: 50 – 1000 Da.

Maximum number of isotopes: 2.

Maximum charged number: 2.

Minimum peak height: 1000 amplitude.

Mass slice width: 0.1 Da.

Smoothing method: Linear weighted moving average.

Smoothing level: 3 scan.

Minimum peak width: 5 scan.

Sigma window value: 0.5.

M/MS abundance cut off: 0 amplitude.

Adduct ion: $[M+H]^+$, $[M+Na]^+$, $[M+K]^+$, $[M-H]^-$, $[M+K-2H]^-$, $[M+Na-2H]^-$.

Alignment -Retention time tolerance: 0.1 min.

-MS1 tolerance: 0.0025 Da.

Compound Discoverver:

The screenshot of workflow from the software.:

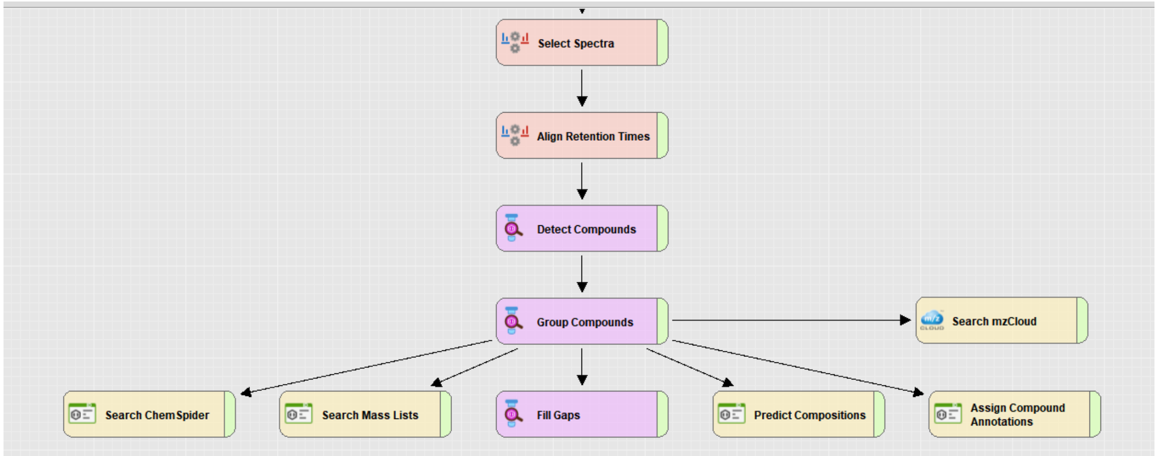

1. Alignment settings:

Alignment model: adaptive curve

Maximum shift (min) :2

Mass Tolerance: 5 ppm

2. Detection settings:

Mass Tolerance: 5 ppm

Min. Peak Intensity: 10,000

Use most intense isotope only: 0.0025 Da.

Chromatographic S/N Threshold: 1.5

Remove Baseline: False

Ions: [M+H]+, [M-H]-

3. Group setting:

Mass tolerance: 5 ppm

RT tolerance (min): 0.2

Minimum Valley (%): 10

Area Integration: Most Common Ion

Area Contribution: 3

CV Contribution: 10

FWHM to Base Contribution: 5

Jaggedness Contribution: 5

Modality Contribution: 5

Zig-Zag Index Contribution: 5

Peak Rating Threshold: 4

Number of files: 1

4. DDA search

Identity Search: Cosine

Match Activation Type: True

Match Activation Energy: Match with Tolerance

Activation Intensity Threshold: True

Similarity Search: Confidence Forward

Match Factor Threshold: 50

Text S4. Instrument analysis

Analysis of the extracts was conducted using a Vanquish UHPLC and Orbitrap Exploris 240 MS (Thermo-Scientific, Waltham, MA). LC separation was achieved with an Ascentis® 3 μm C18 HPLC column (150 × 2.1 mm) (Sigma-Aldrich Supelco, St. Louis, MO) by gradient elution with 5% methanol + 95% HPLC water (A) and 100% methanol (B), both containing 0.1% formic acid at a flow rate of 0.2 mL min^{-1} and column temperature of 45 °C. The gradient method started at 5%B, ramping linearly to 100%B over 15 min, held for 5 min, and returning to starting conditions for column re-equilibration between 20.1 – 25 min.

The compounds in the samples were ionized using a heated electrospray ionization (HESI) probe in both positive (ESI^+) and negative (ESI^-) modes. The Orbitrap MS method used the following global parameters: sheath gas flow = 35; aux gas flow = 10; sweep gas flow = 1; vaporizer temperature = 400 °C; spray voltage = 3300/2000 (positive/negative); S-lens RF = 70%; ion transfer tube temperature = 352 °C. A full MS/data-dependent MS^2 spectra acquisition ($ddMS^2$) method was used with the following scan settings: 90,000/12,000 resolution, normalized AGC target = standard, max injection time = auto, normalized HCD collision energy (%) = 30, 50, 70, full MS scan range of 100-1000 m/z and $ddMS^2$ isolation window of 0.7 m/z and scan number of 10. To confirm the selected chemicals with annotations from Levels 2 and 3, a full MS/product ion scan was conducted for authentic standards and samples.

Text S5. Quality assurance/quality control (QA/QC)

Batch analyses of samples were conducted by running three blanks, i.e., solvent blank, laboratory blank, and field blank. Two solvent blanks were run for each five samples. The QC samples were run at each batch to monitor the stability of the instrument, including RT shifts, mass accuracy, and peak intensity (**Spreadsheet S2**). EPA Phthalate Esters Mix (Sigma-Aldrich, St. Louis, MO) was used for QC with a five-

point calibration curve ranging from 50 – 1000 ng mL^{-1}. After running each batch, a Python package was run to filter the targeted m/z from monoisotopic masses (mass tolerance: 5 ppm) and to check the aligned other values, e.g., m/z, RT, and R values (> 0.5 for all expected compounds) obtained from the linearity (**Spreadsheet S2**). All QC compounds were used for ESI^+ while only dibutyl phthalate was used for ESI^- due to other compounds being poorly charged in negative polarity. In addition to commercial standard mixes for QC, we also made a mixture solution consisting of 17 analytical standards, following the same running and checking procedure as the EPA mixture (Results are shown in **Spreadsheet S2**). The field and laboratory blanks used HPLC water to do the same extraction for the same containers used during the collection procedure. The data collected from all blank samples were used to remove the chemical features of which the abundances were 3 times lower in real samples than those in the blank samples.

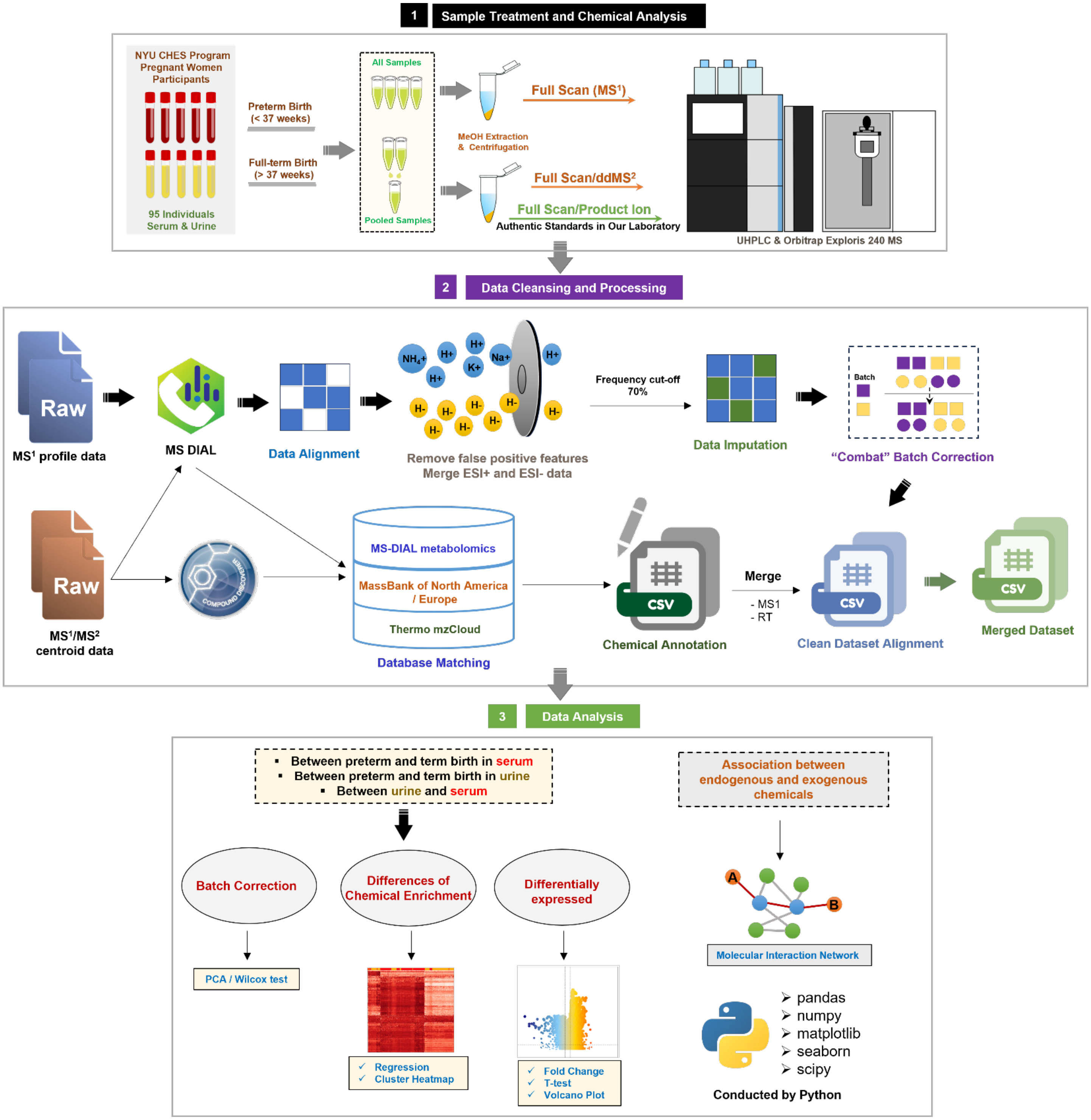

Figure S1. Workflow diagram of sample treatment and chemical analysis, data cleansing and processing, and data analysis for the urine and serum samples collected from 95 pregnant participants in NYU CHES.

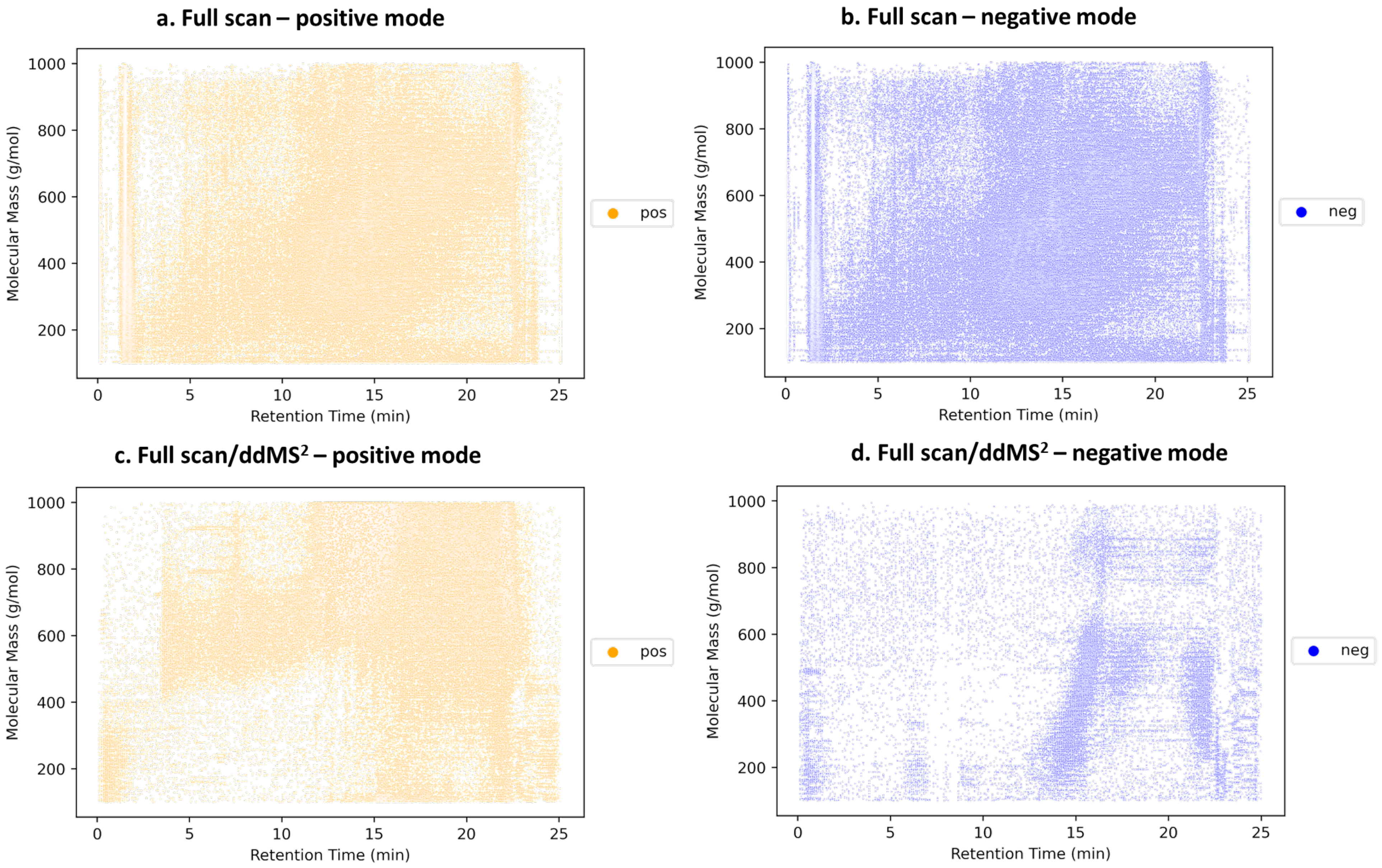
a. Full scan – positive mode
b. Full scan – negative mode
c. Full scan/ddMS² – positive mode
d. Full scan/ddMS² – negative mode
Molecular Mass (g/mol)
Retention Time (min)
pos
neg
0
5
10
15
20
25
200
400
600
800
1000

Figure S2. The highest point or peak of a chromatographic or mass spectrometric signal and abundances (log transformed) of retention times (RT) and molecular mass (g/mol). The plot displays ions from the full scan of the individual samples (**a** and **b**) and the full scan/ddMS^2 of the pooled samples (**c** and **d**) in both positive and negative electrospray ionization modes.

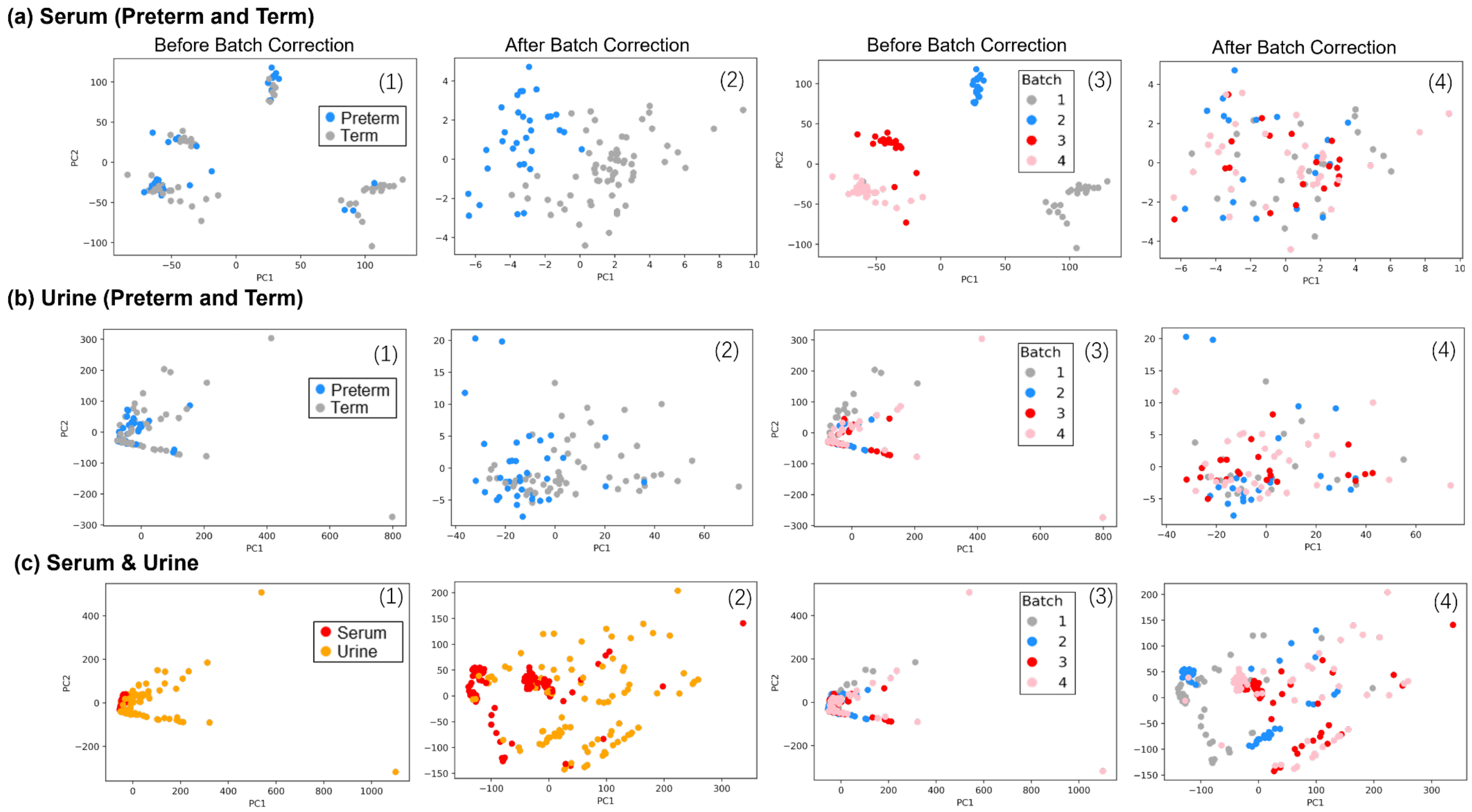
(a) Serum (Preterm and Term)
Before Batch Correction
After Batch Correction
Before Batch Correction
After Batch Correction
(1)
Preterm
Term
(2)
(3)
Batch
1
2
3
4
(4)
PC1
PC2
(b) Urine (Preterm and Term)
(1)
Preterm
Term
(2)
(3)
Batch
1
2
3
4
(4)
PC1
PC2
(c) Serum & Urine
(1)
Serum
Urine
(2)
(3)
Batch
1
2
3
4
(4)
PC1
PC2

Figure S3. Data analysis before and after "Combat" batch correction for the four individual batches in serum (**a**), urine (**b**), and combined urine & serum samples (**c**). The example results demonstrate the following: For preterm and term birth samples in serum or urine (a/b): (**1**) color-labeled principal components analysis (PCA) loadings by preterm and term birth, and (**2**) color-labeled PCA loadings by batch. For combined serum and urine samples (c): (**1**) color-labeled PCA loadings by sample type (serum and urine), and (**2**) color-labeled PCA loadings by batch.

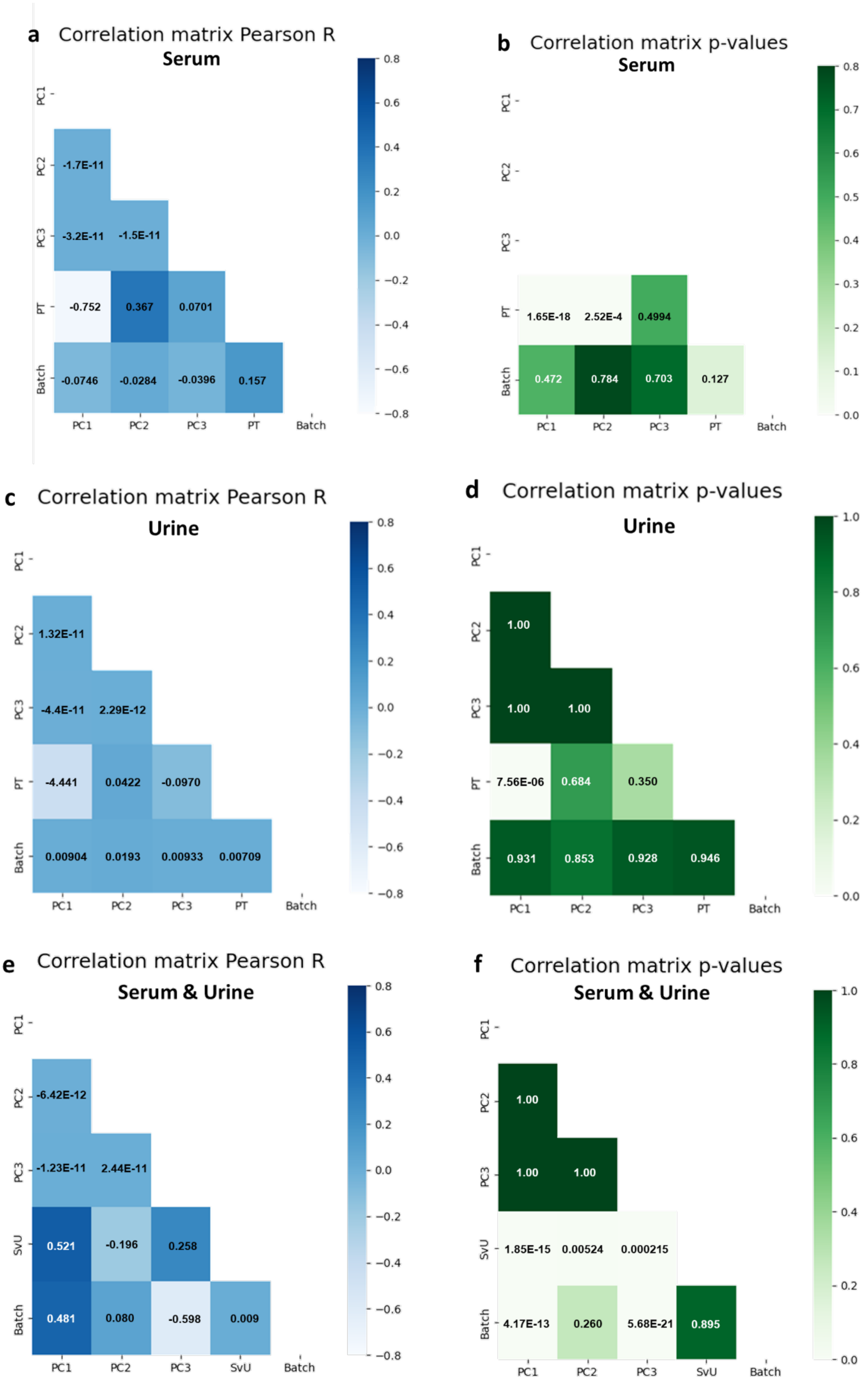
a
Correlation matrix Pearson R
Serum
-1.7E-11
-3.2E-11
-1.5E-11
-0.752
0.367
0.0701
-0.0746
-0.0284
-0.0396
0.157
PC1
PC2
PC3
PT
Batch
0.8
0.6
0.4
0.2
0.0
−0.2
−0.4
−0.6
−0.8
b
Correlation matrix p-values
Serum
1.65E-18
2.52E-4
0.4994
0.472
0.784
0.703
0.127
PC1
PC2
PC3
PT
Batch
0.8
0.7
0.6
0.5
0.4
0.3
0.2
0.1
0.0
c
Correlation matrix Pearson R
Urine
1.32E-11
-4.4E-11
2.29E-12
-4.441
0.0422
-0.0970
0.00904
0.0193
0.00933
0.00709
PC1
PC2
PC3
PT
Batch
0.8
0.6
0.4
0.2
0.0
−0.2
−0.4
−0.6
−0.8
d
Correlation matrix p-values
Urine
1.00
1.00
1.00
7.56E-06
0.684
0.350
0.931
0.853
0.928
0.946
PC1
PC2
PC3
PT
Batch
1.0
0.8
0.6
0.4
0.2
0.0
e
Correlation matrix Pearson R
Serum & Urine
-6.42E-12
-1.23E-11
2.44E-11
0.521
-0.196
0.258
0.481
0.080
-0.598
0.009
PC1
PC2
PC3
SvU
Batch
0.8
0.6
0.4
0.2
0.0
−0.2
−0.4
−0.6
−0.8
f
Correlation matrix p-values
Serum & Urine
1.00
1.00
1.00
1.85E-15
0.00524
0.000215
4.17E-13
0.260
5.68E-21
0.895
PC1
PC2
PC3
SvU
Batch
1.0
0.8
0.6
0.4
0.2
0.0

Figure S4. The dataset analysis was conducted after processing with the "Combat" batch correction to examine correlations and statistically significant differences among principal component (PC) loadings, batch, and sample type. The batch correction was applied for preterm and term birth samples across four batches in both urine and serum, as well as for combined urine and serum samples in four batches. The Pearson R values and p-values were reported for urine (**a** and **b**), serum (**c** and **d**), and combined serum & urine (**e** and **f**). PT: preterm and term birth sample types, SvU: serum and urine sample types.

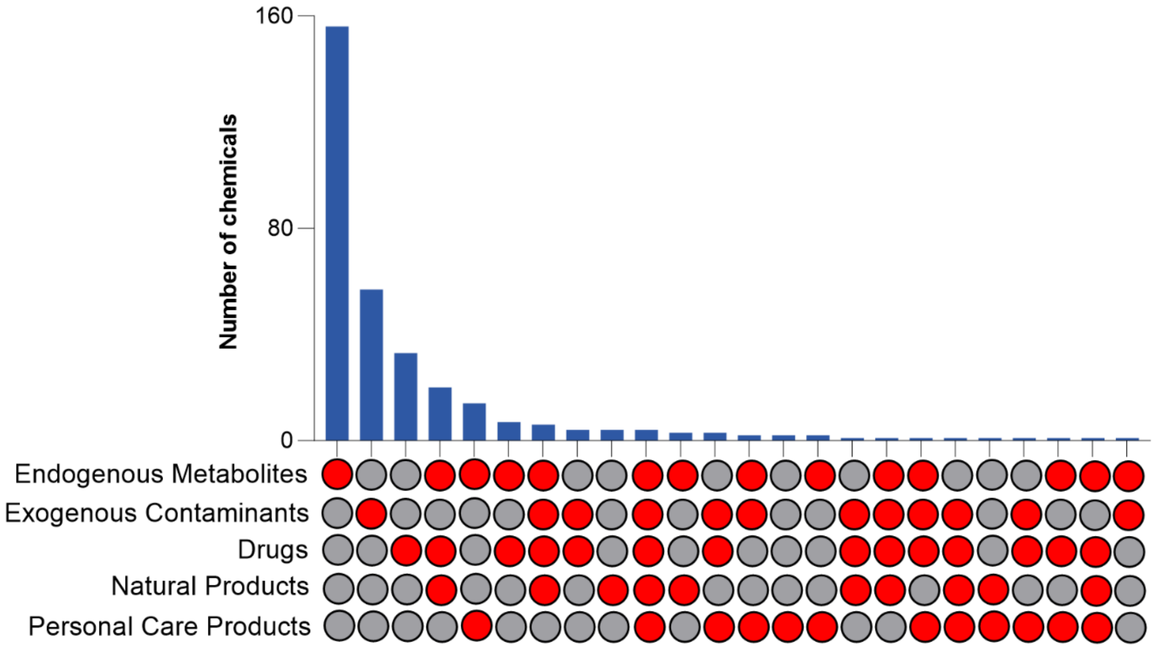

Figure S5. Level 2 chemicals annotated as endogenous metabolites, exogenous contaminants, drugs, natural products, and personal care products. The bar chart shows the full tally of annotated chemicals in each specific category, plotting with the decreasing number of chemicals. The red and grey balls represent the annotated category and non-annotated category, respectively.

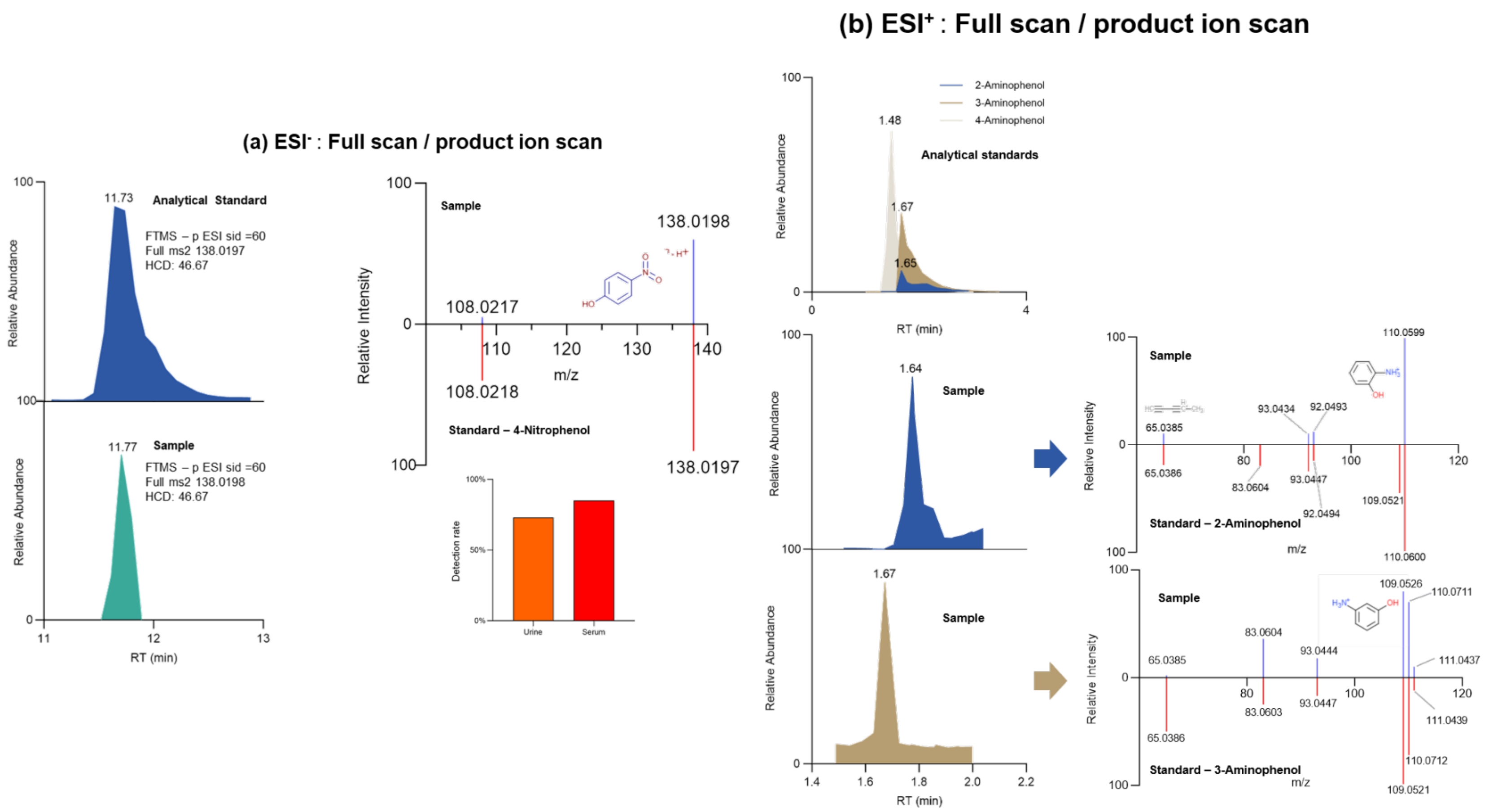

Figure S6. Examples of compounds using authentic standards detected by UHPLC-Orbitrap MS with ESI- (**a**) and ESI+ (**b**) polarities. The chromatographic plot of 4-nitrophenol and aminophenol isomers in the authentic standards and real samples. The compounds were initially selected by comparing different databases and were further confirmed by authentic standards. The differential plot of the deconvoluted spectrum between real samples and authentical standards.

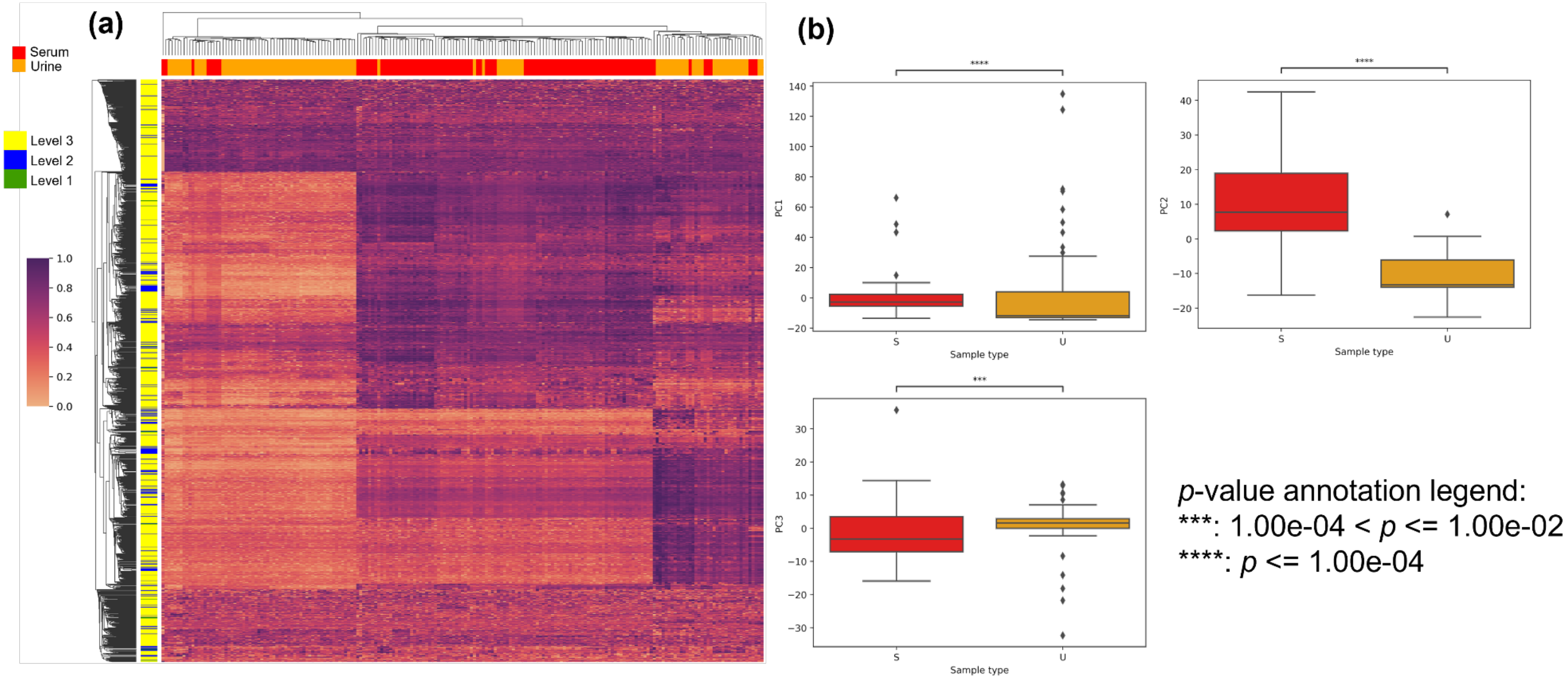

Figure S7. Clustering heatmap of chemical abundances after batch effect correction for serum (S) and urine samples (U) (**a**) and the boxplot for the significant difference ($p < 0.001$) of PCs 1-3 between serum and urine samples using Mann-Whitney-Wilcoxon test two-sided with Bonferroni correction (**b**). The bottom and top of boxes represent the 25th and 75th percentiles, the error bars denote 10th to 90th percentiles, the solid line means the median value. The total number of chemical features (combing ESI$^+$ and ESI$^-$) is 1524 with the annotation levels 1-3 from the classification of Schymanski, et al. [3].

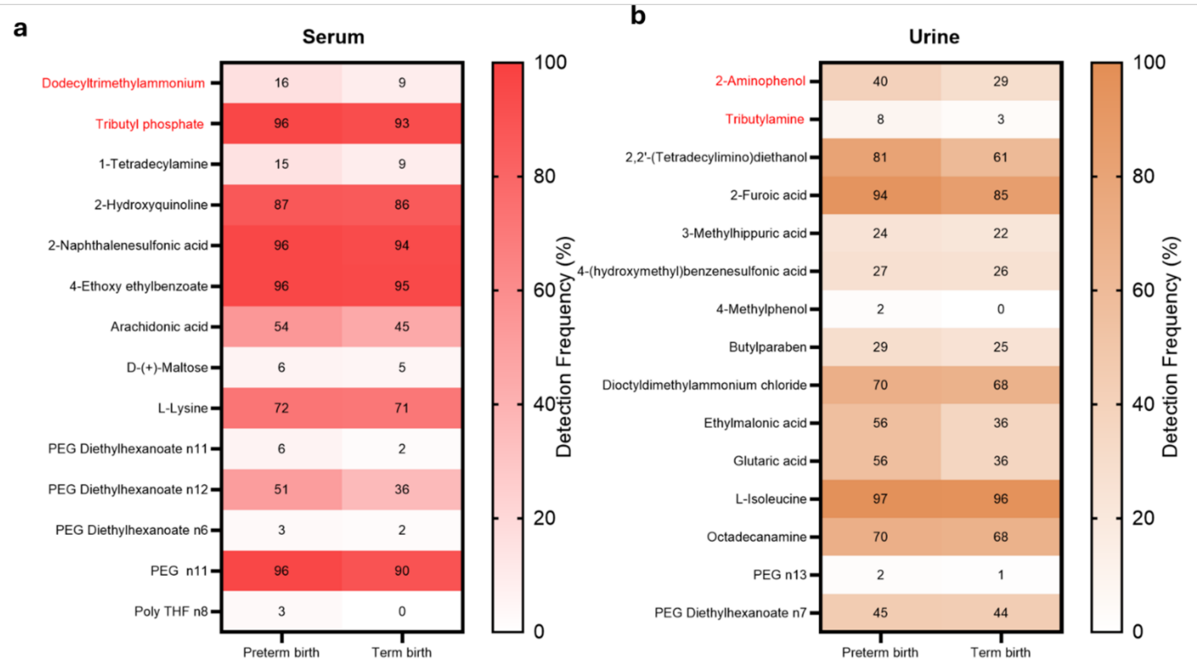

Figure S8. The detection frequency (%) of annotated chemicals (Levels 1 and 2) classified as exogenous contaminants in preterm and term birth samples: chemicals with higher detection frequency in preterm birth only serum (**a**); and in only urine (**b**). The chemical names in red represent the confirmed chemicals (Level 1) by the authentic standards.

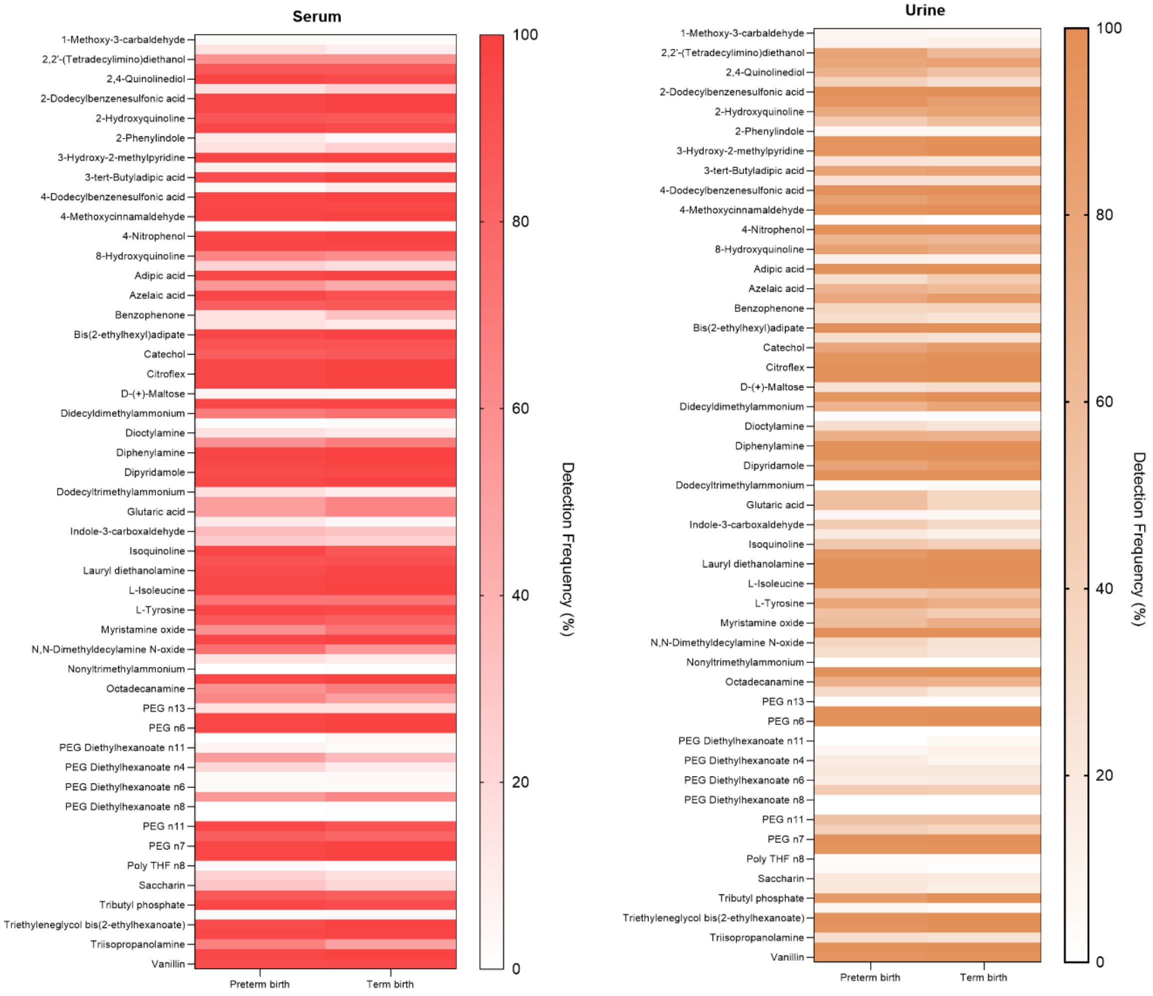

Figure S9. The heatmap of detection frequency (%) for annotated chemicals (Levels 1 and 2) classified as exogenous contaminants in preterm and term birth in serum and urine samples, respectively. The specific number of frequencies is shown in the spreadsheet S1.

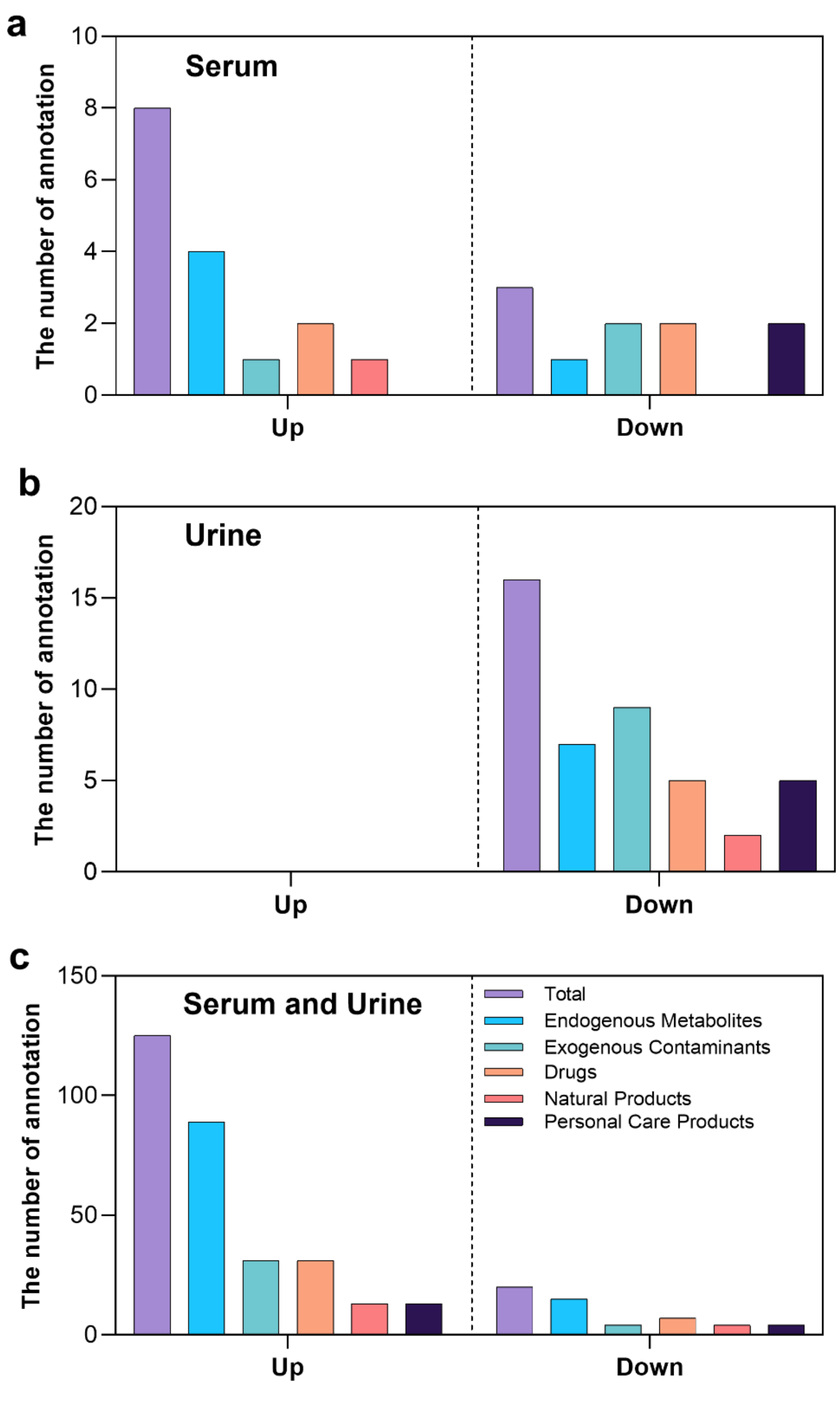

Figure S10. The bar chart of the number of chemicals that were successfully annotated, located in the down- ($p < 0.05$, $\log_2$fold < -1.2) and up- regulated areas ($p < 0.05$, $\log_2$fold >1.2) of the serum vs. urine samples / preterm and term birth samples in serum or urine from the volcano plot (Figure 3).

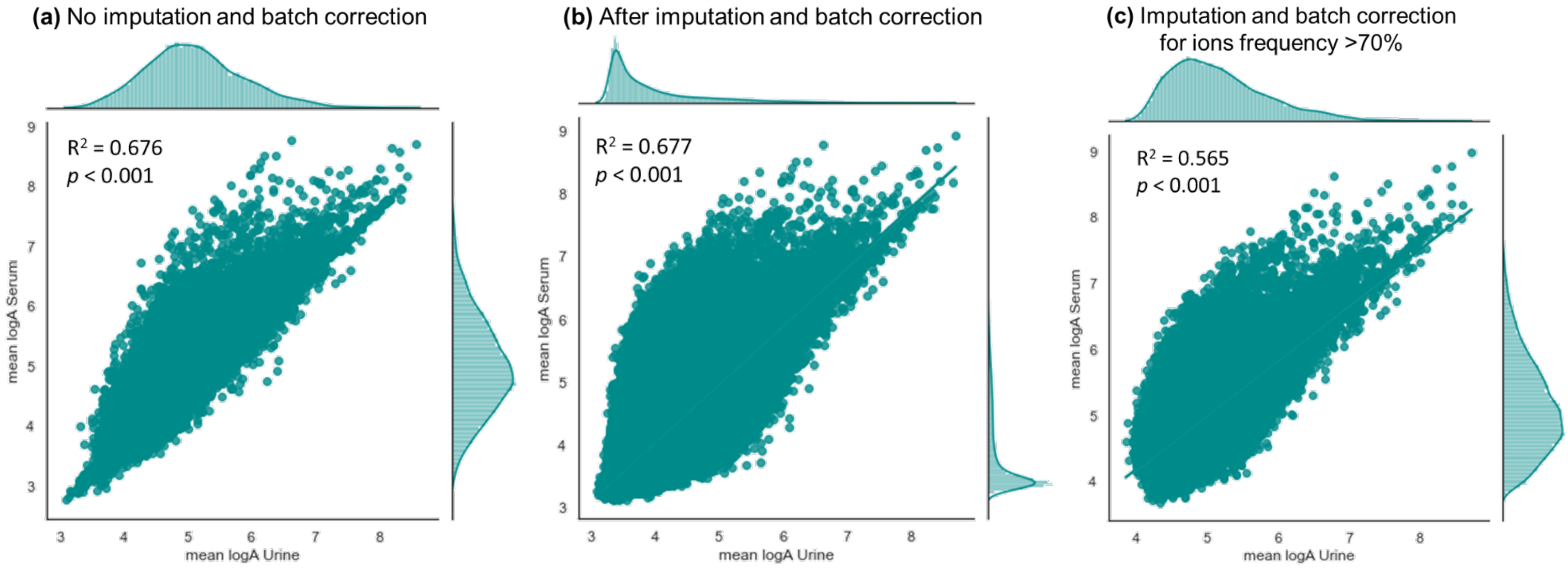

Figure S11. Linear regression correlation analysis between urine and serum abundances in logarithm scale (n = 190 urine and serum). The results show: (**a**) all original dataset before imputation and batch correction, (**b**) all dataset after imputation and batch correction, and (**c**) the ions filtered by the cut-off frequency of 70% for imputation and batch correction.

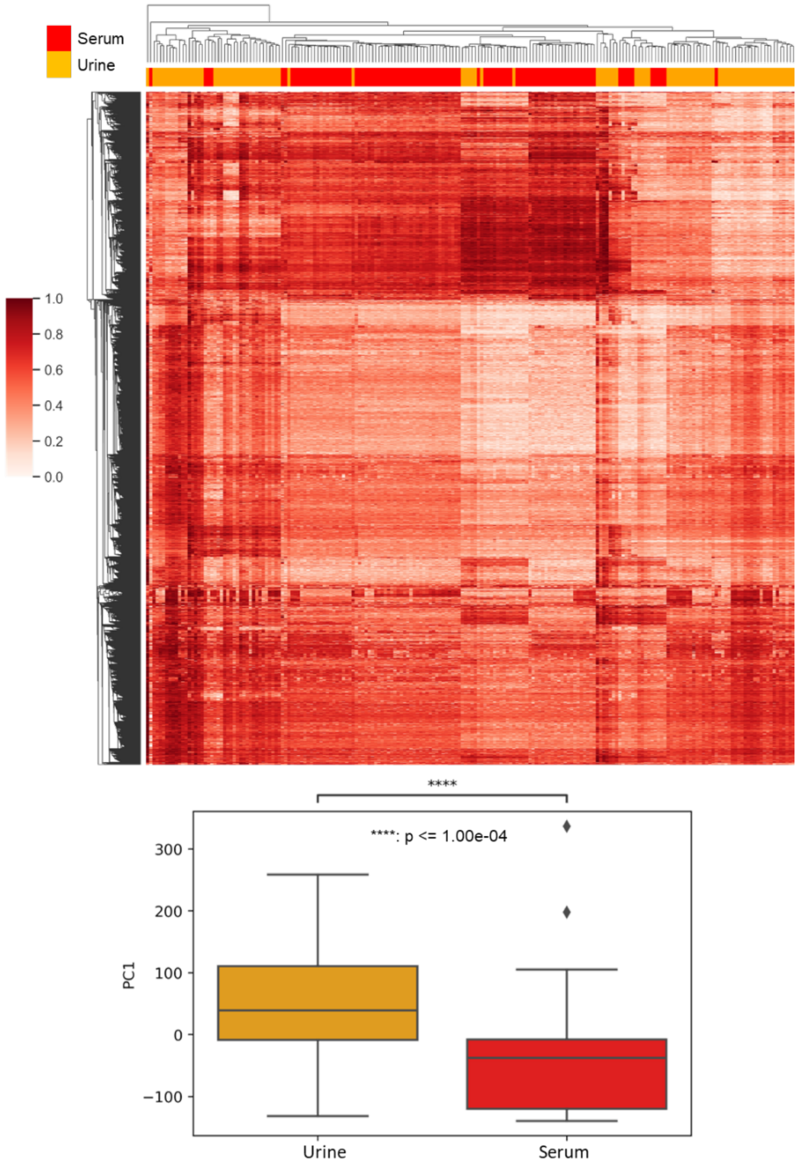

Figure S12. Clustering heatmap after batch effect correction for serum and urine samples. The chemical features reveal the differential enrichment between serum and urine samples after multiple testing correction (Benjamini-Hochberg test, 5% false discovery rate). For the differential enrichment between serum and urine samples, 26,038 out of 37,270 chemical features exhibited significant differences ($p < 0.05$). The boxplots show the statistical difference of principal component 1 (PC1) between urine and serum samples using the Mann-Whitney-Wilcoxon test (two-sided) with Bonferroni correction. The bottom and top of the boxes represent the 25th and 75th percentiles, the error bars denote the 10th to 90th percentiles, and the solid line indicates the median value.

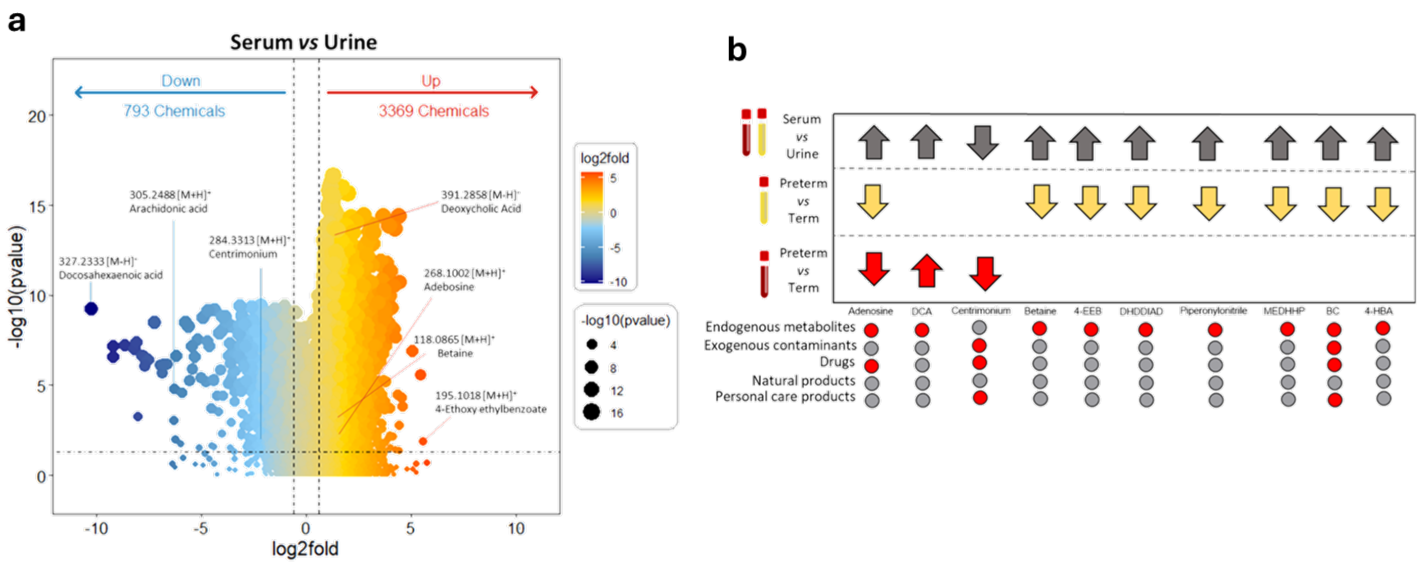

Figure S13. The volcano plot of the log-transformed ratios and corresponding p-values of chemical features with a cut-off frequency of 70% from ESI^+ and ESI^- modes illustrates the data: the statistical differences in chemical features between serum and urine (**a**). The horizontal dashed line indicates the cutoff for the log *p*-value ($p < 0.05$), and the vertical dashed lines indicate the cutoff for fold change (Log_2 fold change = 1.2). The arrow graph (**b**) indicates the regulation status of the same annotated chemical across different volcano plots (**Figure 3a&b** and **this figure a**). Up arrows represent up-regulated areas, while down arrows indicate down-regulated areas. Red balls denote annotated categories, and grey balls represent non-annotated categories. DCA: Deoxycholic Acid, 4-EEB: 4-Ethoxy ethylbenzoate, 4-HBA: 4-Hydroxybenzaldehyde, MEDHHP: Methyl 2-[4-ethenyl-2,6-dihydroxy-3-(3-hydroxyprop-1-en-2-yl)-4-methylcyclohexyl]prop-2-enoate, DHDDIAD: 1,4-dihydroxy-1,4-dimethyl-7-(propan-2-ylidene)-decahydroazulen-6-one, BC: Benzoic Acid.

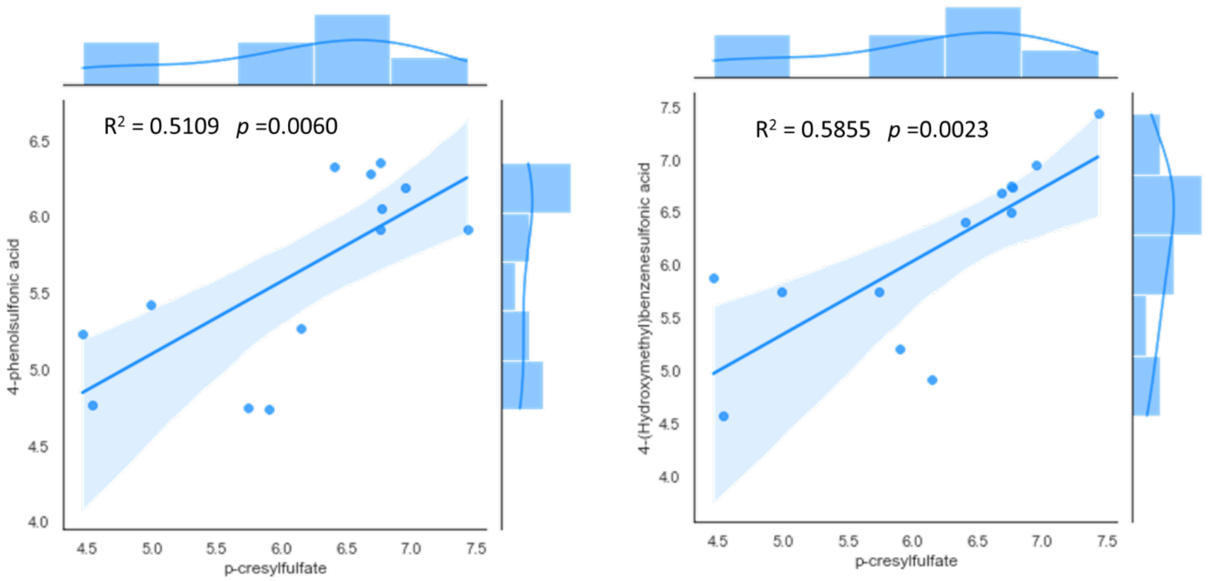

Figure S14. Correlation between p-cresylfulfate and 4-(hydroxymethyl)benzenesulfonic acid, and p-cresylfulfate and 4-phenolsulfonic acid in logarithm scale (*n* = 95 serum).

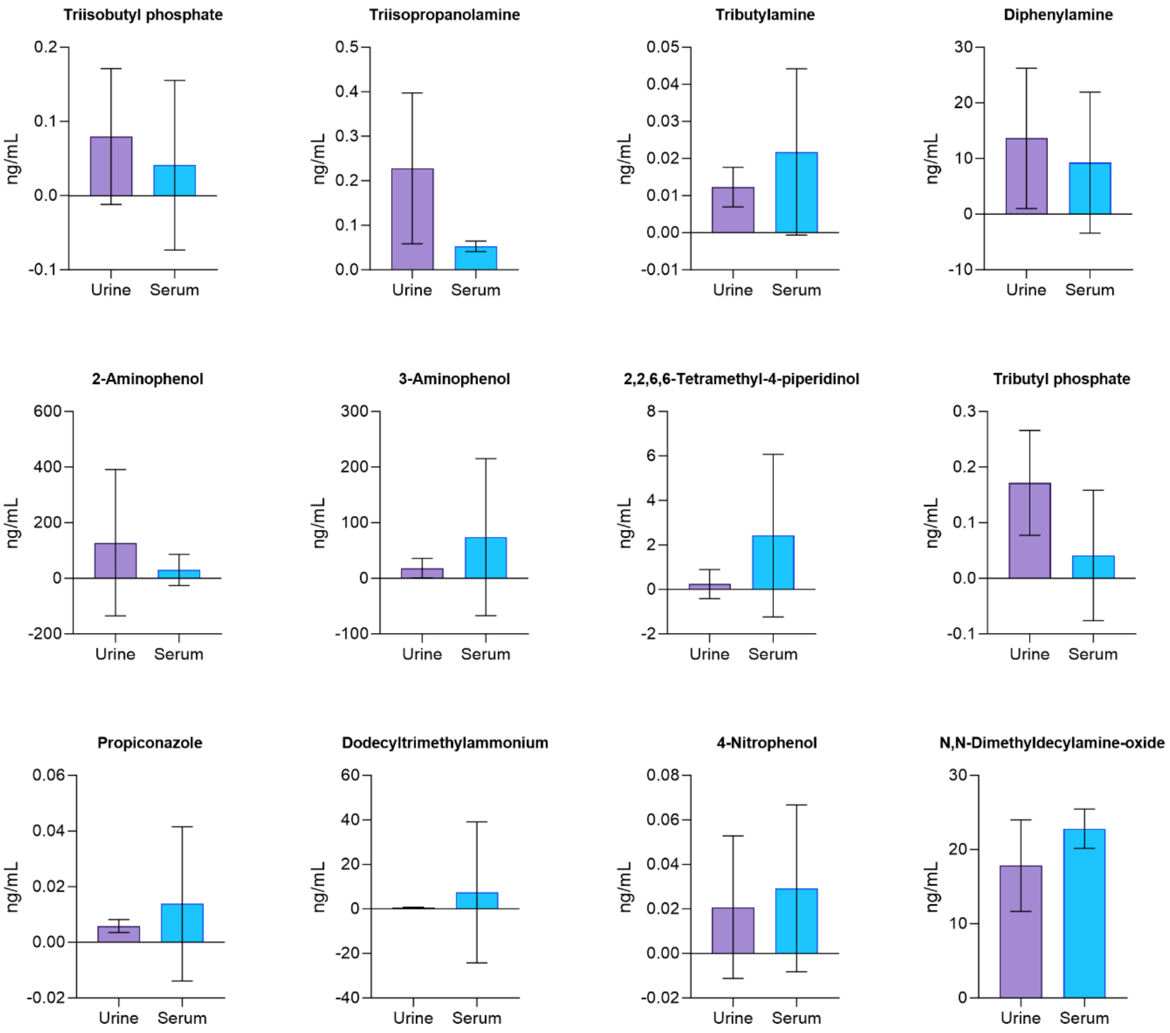

Figure S15. The semi-quantification of confirmed compounds (level 1) in urine and serum samples based on the integral peak areas of 500 ng/mL analytical standards.

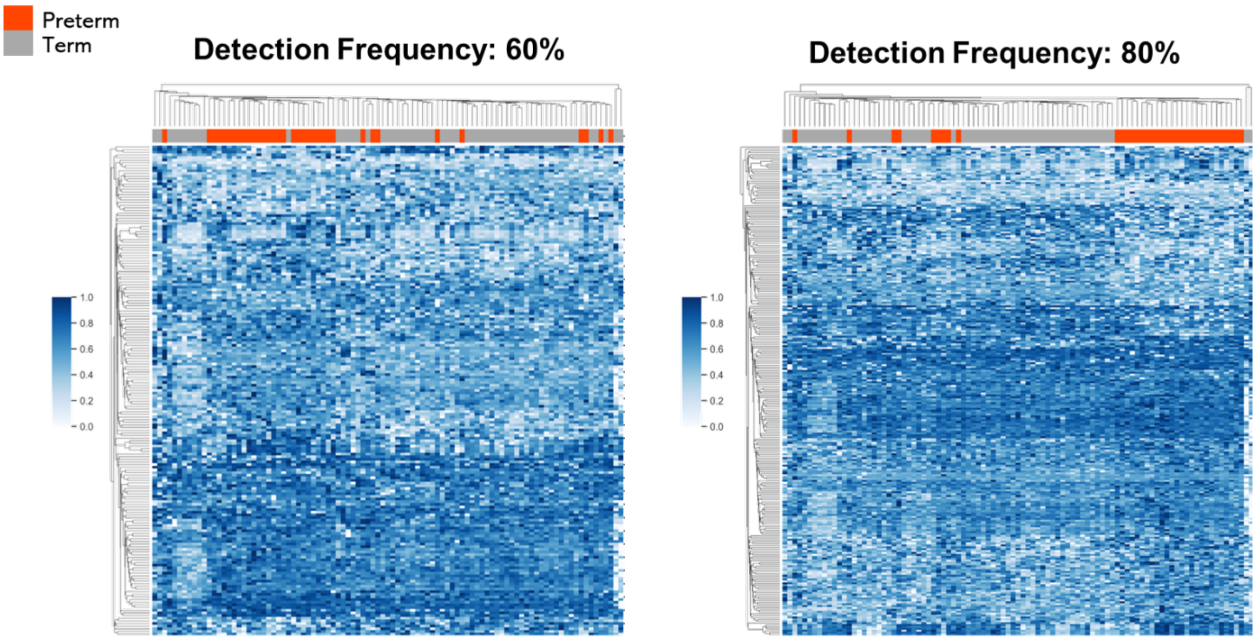

Figure S16. Clustering heatmap after batch effect correction (between preterm and term birth) for serum samples. The chemical features reveal the differential enrichment in preterm versus term births among serum with the cut-off detection frequencies of 60% and 80% after multiple testing correction (Benjamini-Hochberg test, 5% false discovery rate). For the differential enrichment in preterm versus term birth samples, 1,791 out of 43,450 chemical features in a detection frequency cut-off of 60% and 1,214 out of 25,323 in a detection frequency cut-off of 80% showed significant differences ($p < 0.05$).

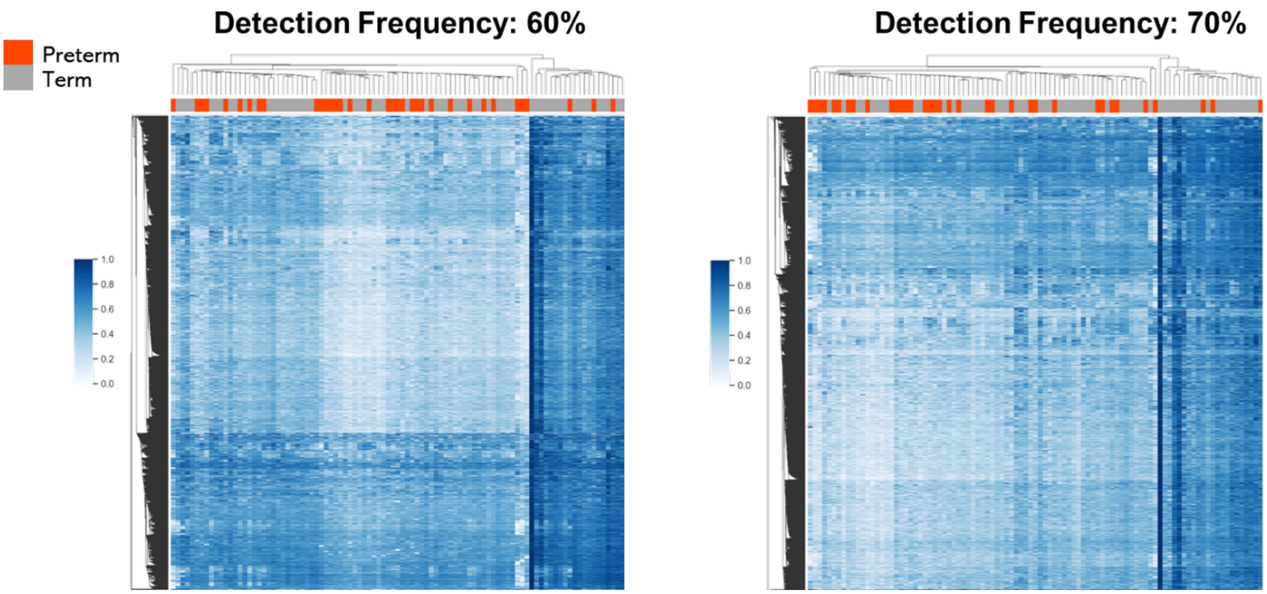

Figure S17. Clustering heatmap after batch effect correction (between preterm and term birth) for urine samples. The chemical features reveal the differential enrichment in preterm versus term births among serum with the cut-off detection frequencies of 60% and 80% after multiple testing correction (Benjamini-Hochberg test, 5% false discovery rate). For the differential enrichment in preterm versus term birth samples, 9,518 out of 49,350 chemical features in a detection frequency cut-off of 60% and 8,398 out of 29,448 in a detection frequency cut-off of 80% showed significant differences ($p < 0.05$).

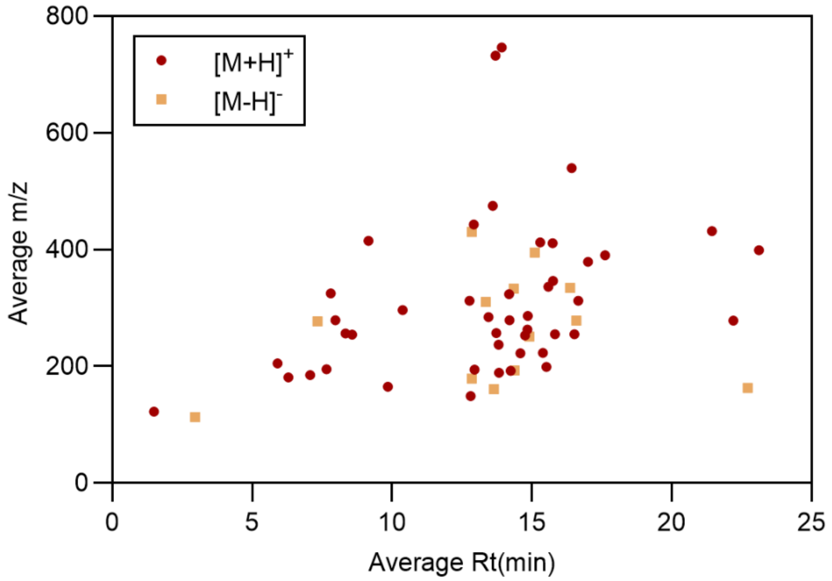

Figure S18. The average m/z values of quality control (QC) compounds at their corresponding average retention times (RT, min) across the LC-MS running time.

References

1 Cone, E. J. *et al.* Normalization of Urinary Drug Concentrations with Specific Gravity and Creatinine. *Journal of Analytical Toxicology* **33**, 1-7 (2009). https://doi.org/10.1093/jat/33.1.1

2 Hou, H. *et al.* LC-MS-MS Measurements of Urinary Creatinine and the Application of Creatinine Normalization Technique on Cotinine in Smokers' 24 Hour Urine. *Journal of Analytical Methods in Chemistry* **2012**, 245415 (2012). https://doi.org/10.1155/2012/245415

3 Schymanski, E. L. *et al.* Identifying Small Molecules via High Resolution Mass Spectrometry: Communicating Confidence. *Environmental Science & Technology* **48**, 2097-2098 (2014). https://doi.org/10.1021/es5002105
